# NEDD4-family E3 ligase dysfunction due to *PKHD1/Pkhd1* defects suggests a mechanistic model for ARPKD pathobiology

**DOI:** 10.1038/s41598-017-08284-4

**Published:** 2017-08-10

**Authors:** Jun-ya Kaimori, Cheng-Chao Lin, Patricia Outeda, Miguel A. Garcia-Gonzalez, Luis F. Menezes, Erum A. Hartung, Ao Li, Guanqing Wu, Hideaki Fujita, Yasunori Sato, Yasuni Nakanuma, Satoko Yamamoto, Naotsugu Ichimaru, Shiro Takahara, Yoshitaka Isaka, Terry Watnick, Luiz F. Onuchic, Lisa M. Guay-Woodford, Gregory G. Germino

**Affiliations:** 1 Department of Nephrology, Osaka University Graduate School of Medicine, 2-2 Yamadaoka, Suita, Osaka 565-0871 Japan; 2 Department of Advanced Technology of Transplantation, Osaka University Graduate School of Medicine, 2-2 Yamadaoka, Suita, Osaka 565-0871 Japan; 3 National Institute of Diabetes and Digestive and Kidney Diseases, National Institutes of Health, Bldg 31, 9A52, 31 Center Drive, Bethesda, MD20892 USA; 4 University of Maryland School of Medicine, Division of Nephrology, 22S Green St, Baltimore, MD21201 USA; 5 Laboratorio de Investigacion en Nefroloxia, Complexo Hospitalario Universitario de Santiago, Travesía de Choupana, s/n 15706 Santiago de Compostela Spain; 6 Division of Nephrology, Children’s Hospital of Philadelphia, Perelman School of the University of Pennsylvania, 3401 Civic Center Boulevard, CTRB 9207, Philadelphia, PA 19104 USA; 7 Center of Translational Cancer Research and Therapy, State Key Laboratory of Molecular Oncology, Cancer Hospital and Institute, Chinese Academy of Medical Sciences and Peking Union Medical College, Beijing, 100021 China; 8 Graduate School of Pharmaceutical Science, Nagasaki International University, 2825-7 Huis Ten Bosch-Cho, Sasebo, Nagasaki 859-3298 Japan; 9 Departments of Human Pathology, Kanazawa University Graduate School of Medicine, 13-1 Takara-cho, Kanazawa, 920-8640 Japan; 10 Department of Pathology, Shizuoka Cancer Center, 1007 Shimonagakubo, Nagaizumi-cho, Sunto-gun, Shizuoka, 411-8777 Japan; 11 Department of Medicine, Division of Nephrology, University of Sao Paulo, Dr. Arnald Ave, 455-Cerqueira Cesar, Sao Paulo, CEP01246903-903 Brazil; 12 Children’s National Health System, 6th Floor Main Hospital, Center 6, 111 Michigan Ave NW, Washington, DC 20010 USA; 13 Johns Hopkins University School of Medicine, Department of Medicine, Division of Nephrology, 1830 East Monument St., Suite 416, Baltimore, MD21287 USA

## Abstract

Autosomal recessive polycystic kidney disease (ARPKD) is an important childhood nephropathy, occurring 1 in 20,000 live births. The major clinical phenotypes are expressed in the kidney with dilatation of the collecting ducts, systemic hypertension, and progressive renal insufficiency, and in the liver with biliary dysgenesis, portal tract fibrosis, and portal hypertension. The systemic hypertension has been attributed to enhanced distal sodium reabsorption in the kidney, the structural defects have been ascribed to altered cellular morphology, and fibrosis to increased TGF-β signaling in the kidney and biliary tract, respectively. The pathogenic mechanisms underlying these abnormalities have not been determined. In the current report, we find that disrupting *PKHD1* results in altered sub-cellular localization and function of the C2-WWW-HECT domain E3 family of ligases regulating these processes. We also demonstrate altered activity of RhoA and increased TGF-β signaling and ENaC activity. Linking these phenomena, we found that vesicles containing the *PKHD1/Pkhd1* gene product, FPC, also contain the NEDD4 ubiquitin ligase interacting protein, NDFIP2, which interacts with multiple members of the C2-WWW-HECT domain E3 family of ligases. Our results provide a mechanistic explanation for both the cellular effects and *in vivo* phenotypic abnormalities in mice and humans that result from *Pkhd1/PKHD1* mutation.

## Introduction

Autosomal recessive polycystic kidney disease (ARPKD; MIM 263200) is an inherited disorder of the kidney and liver that affects 1/20,000 live births. A substantial fraction of affected children dies within the first year of life, often due to complications of hypoplastic lung disease. Most survivors of the neonatal period develop systemic hypertension^1^, with about 25–30% developing ESRD in the first decade of life^2^. Cysts mostly are derived from fusiform dilatations of the collecting ducts. The cyst-lining epithelia cells are reported to have increased rates of sodium absorption^3, 4^, a factor that likely contributes to the high prevalence of hypertension in this population. Renal fibrosis is a later feature and presumed to be the result of increased TGF-β signaling.

Liver disease is a universal feature of this disorder, characterized by a ductal plate malformation (DPM) that leads to increased number of irregularly shaped and dilated biliary ductules, with variable degrees of associated fibrosis. The DPM defect with its associated fibrosis often results in severe portal hypertension^5^. The pathogenic mechanisms underlying the liver disease phenotype are largely unknown. Several studies suggest that the liver fibrosis is the result of increased TGF-β signaling in cholangiocytes. Analysis of tissue sections and cultured cells from rodent *Pkhd1*-mutant models have found increased expression of TGF-β, its receptor and target gene products as well as enhanced responsiveness to TGF-β stimulation^6, 7^. The evidence showing increased TGF-β activity is mostly circumstantial, however, and the mechanisms responsible for the increased TGF-β activity in *Pkhd1* mutants have not been identified.

All typical forms of ARPKD result from mutations of a single gene, *PKHD1* ^8–11^. Mutations have been identified involving most of the 67 exons that encode the gene’s longest open reading frame^12^. The gene encodes a novel, type I membrane protein of 4074 amino acids (aa) called fibrocystin/polyductin complex (FPC) that contains multiple Ig-like, plexin, transcription factor domains (IPT) and parallel beta-helix repeats (PbH1) in its long, extracellular N-terminus^9, 10^. The protein localizes to the primary cilium and other sub-cellular locations where it may function as a receptor for an unidentified ligand^13–17^. It also undergoes a complicated Notch-like pattern of proteolytic processing whose activity may be regulated, possibly by ligand-binding^18, 19^. This process results in a shed ectodomain that also may have biological activity. The *PKHD1* gene has transcriptional complexity^10, 20^. The functions of the full-length protein, its various isoforms and proteolytic products remain poorly defined.

In the current study, we identified novel functional relationships between FPC and members of the C2-WWW-HECT domain E3 family of ubiquitin ligases. Disrupting *Pkhd1* function alters both the sub-cellular localization and function of these ligases, resulting in increased activity of TGF-β in biliary epithelial cells and ENaC in collecting duct cells. In characterizing these findings, we determined that vesicles containing FPC also contained Ndfip2, a ubiquitin ligase interacting protein that has been implicated in trafficking and regulating the Nedd4 ubiquitin ligase family^21, 22^. Our results provide a mechanistic explanation for the cellular effects of *Pkhd1* in kidney and liver and suggest mechanisms that underlie the *in vivo* phenotypic abnormalities evident in human disease.

## Results

### Changes in *PKHD1* activity result in altered levels and function of the Rho GTPase family protein, RhoA

**Figure 1:**
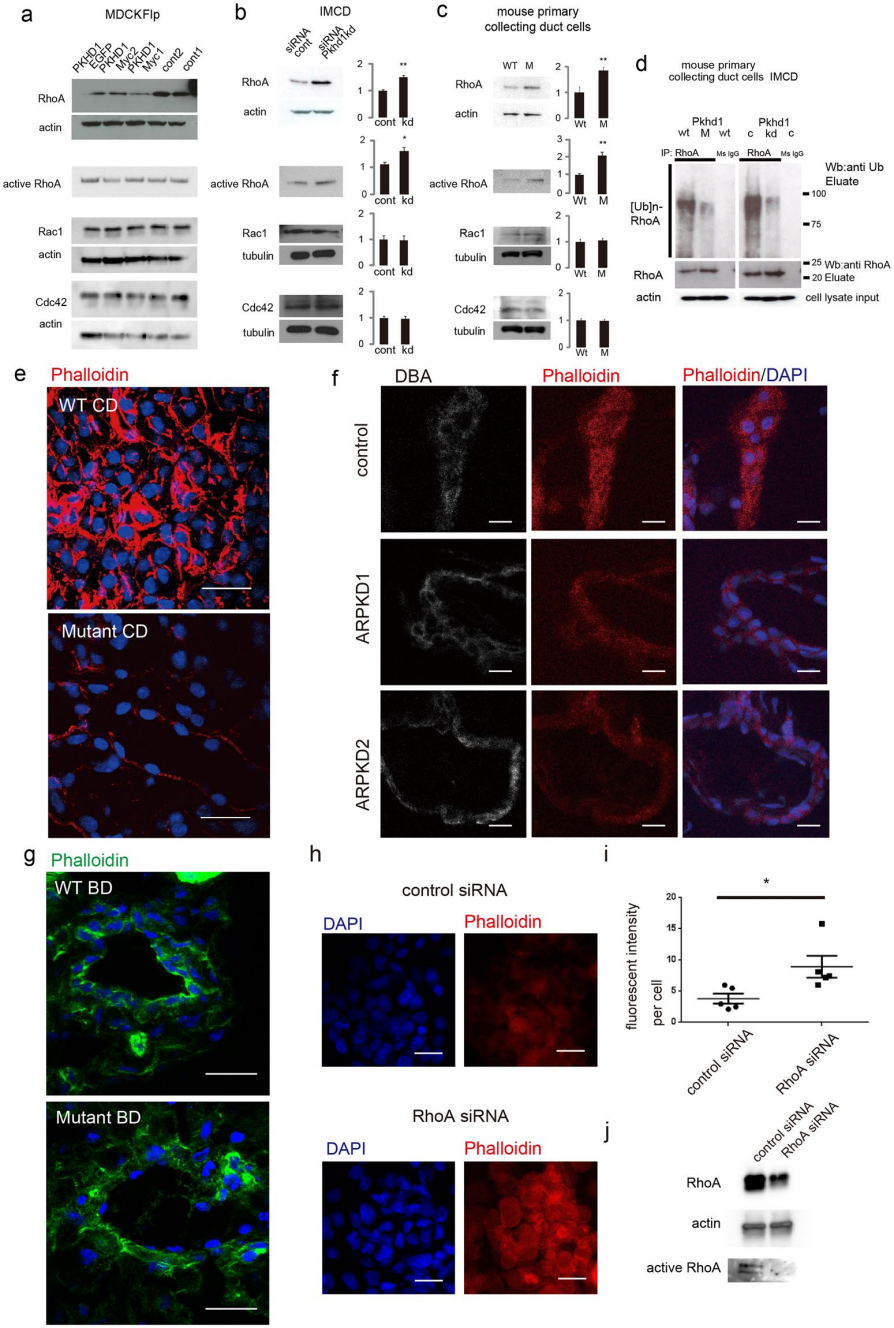
Fibrocystin/Polyductin (FPC) regulates RhoA levels and function. (**a**) Immunoblots of total cell lysates of multiple independently-derived *PKHD1*-expressing and pcDNA5 vector control (cont) Flp-In MDCK cell lines probed for RhoA, active RhoA, Rac1 and Cdc42. The blots were stripped and re-probed for actin as a loading control (except the active RhoA blot). (**b**) On the left are immunoblots of cell lysates from IMCD cell lines stably expressing siRNA targeting either *Pkhd1* (kd) or a random sequence (cont). The bar graphs on the right provide the quantitative analysis of three independent experiments. The level of expression of each Rho GTPase was determined relative to that of actin or tubulin, and the values for the control cell line was arbitrarily set at one. For the active RhoA blot, equal amounts of cell lysate were loaded. Values represent mean ± s.d. **P < 0.01. (**c**) Same as in panel “b” except primary cultures of collecting duct cells isolated from wild type controls (WT) or *Pkhd1* ^*del3-4/del3-4*^ (M) were the source material. Values represent the mean ± s.d. of three experiments, **P < 0.01. (**d**) RhoA was immunoprecipitated from cell lysates of primary cultures of wild type (WT) and *Pkhd1* ^del3-4/del3-4^ (M) collecting duct cells and control (C) and *Pkhd1*-silenced IMCD (kd) cells and then immunoblotted for ubiquitin (top panel) and RhoA (middle panel). Mouse IgG was used as a negative control (IgG). Equal amounts of cell lysate input were immunoblotted for actin (bottom panel).(**e**) Kidney of control (WT) and littermate *Pkhd1* ^del3-4/del3-4^ (Mutant) mice stained with phalloidin (red) and DAPI (blue). Scale bars, 20 μm. (**f**) Kidney specimens from human control (control) and two different ARPKD donors (ARPKD1, ARPKD2) stained for Dolichos Biflorus Agglutinin (DBA), a marker for collecting ducts (grey), phalloidin (red) and DAPI (blue). Scale bars, 10μm. (**g**) Bile ducts of control (WT BD) and littermate *Pkhd1* ^del3-4/del3-4^ (Mutant BD) mice stained with phalloidin (green) and DAPI (blue). Scale bars, 20 μm. (**h**) Cholangiocyte cell line derived from PCK rat treated with either scrambled control or RhoA siRNA and stained with DAPI (blue) and phalloidin (red). Scale bars, 20 μm. (**i**)Scatter plot of the fluorescent intensity for studies represented in “h”. Asterisk denotes p < 0.05. (**j**)Representative western blot of lysate from cell cultures described in “h” and probed for RhoA and beta-actin as a loading control. Active RhoA levels are displayed in bottom panel. The full-length blots of cropped images in a-d and j are included in Supplementary Figure S7.

In characterizing a series of MDCK cell lines with inducible expression of *PKHD1* ^18^, we found that the *PKHD1*+ cell lines had different cellular morphology at confluence than vector controls (Supplementary Fig. S1a,b,c). Since altered cellular morphology is a characteristic feature of cystic epithelial cells, we examined possible mechanisms that might mediate these effects. The Rho small GTPase family is known to play an essential role in regulating cell architecture, though the relationship between the activity of specific Rho family members and their associated cellular effects are complex and both context- and cell-type dependent^6, 23–27^. We therefore evaluated the expression of RhoA, Rac1 and Cdc42 and found that RhoA expression was consistently lower in the *PKHD1*+ cell lines than in controls while levels of Rac1 and Cdc42 did not differ (Fig. 1a). We confirmed the inverse relationship between *Pkhd1* activity and RhoA levels in two independent cell culture models with reduced FPC activity: a) an IMCD cell line with *Pkhd1* expression stably reduced by siRNA^28^, and b) primary collecting duct cells isolated from a previously described *Pkhd1* mouse mutant (*Pkhd1* ^*del3-4/del3-4*^) that recapitulates many features of human ARPKD^29^. The two studies yielded similar results (Fig. 1b,c). RhoA was increased in cells with reduced *Pkhd1* expression whereas levels of Rac1 and Cdc42 were unchanged. Collectively, these data demonstrate an inverse relationship between *PKHD1/Pkhd1* expression and RhoA protein levels. The levels of active RhoA in *PKHD1* stably expressing MCKD cells were not different from those in control MCKD cells (Fig. 1a), whereas the quantities of active RhoA in *Pkhd1* kd or *Pkhd1* mouse mutant cells were increased (Fig. 1b,c).

RhoA protein levels are regulated by the protein degradation pathway rather than by changes in gene expression^30^. Smurf1, a member of the C2-WWW-HECT domain E3 family of ubiquitin ligases, is a major regulator of RhoA levels and thereby an important modulator of cell polarity^31^ and epithelial cell plasticity^32^. If the observed changes in RhoA levels are due to changes in Smurf1 activity, one would expect to see inverse changes in RhoA ubiquitination. The *Pkhd1* ^*del3-4/del3-4*^ collecting duct cells and the *Pkhd1* siRNA-silenced IMCD cell line with their relatively higher RhoA levels would be expected to have reduced levels of ubiquitinated RhoA compared to their respective controls. We tested for this possibility by immunoprecipitating RhoA and immunoblotting for ubiquitin. The immunoprecipitation study revealed that in both the *Pkhd1* ^*del3-4/del3-4*^ collecting duct cells and in the *Pkhd1* siRNA-silenced IMCD cell line levels of ubiquitinated RhoA were reduced (Fig. 1d).

RhoA is a major regulator of the cytoskeleton by controlling F-actin architecture. We therefore examined F-actin patterns in tissue specimens of *Pkhd1* mutants. In kidney, F-actin was present diffusely in the collecting duct (CD) cells of both normal mouse and human kidney but at greatly decreased levels in *Pkhd1* ^*del3-4/del3-4*^ mutant and human ARPKD kidney specimens (Fig. 1e,f; Supplementary Fig. S2a). In wild type liver, F-actin predominantly localized to the sub-cortical region of biliary ductules (BD) whereas in the *Pkhd1* ^*del3-4/del3-4*^ mutants F-actin levels were greatly decreased and expressed more diffusely within the cell (Fig. 1g; Supplementary Fig. S2b,c). To investigate whether increased RhoA protein abundance in ARPKD tissues really induces disruption of F-actin formation, we used siRNA to decrease RhoA levels in a cholangiocyte cell line derived from PCK rats, a Sprague-Dawley strain (Crj:CD/SD) with a spontaneous *Pkhd1* mutation^33, 34^. Treatment with RhoA siRNA resulted in increased levels of F-actin (Fig. 1h–j), suggesting a role for increased RhoA protein expression in the cell structural changes observed in ARPKD tissues.

### Changes in *PKHD1* function affect the sub-cellular localization of Smurf1 and Smurf2

**Figure 2:**
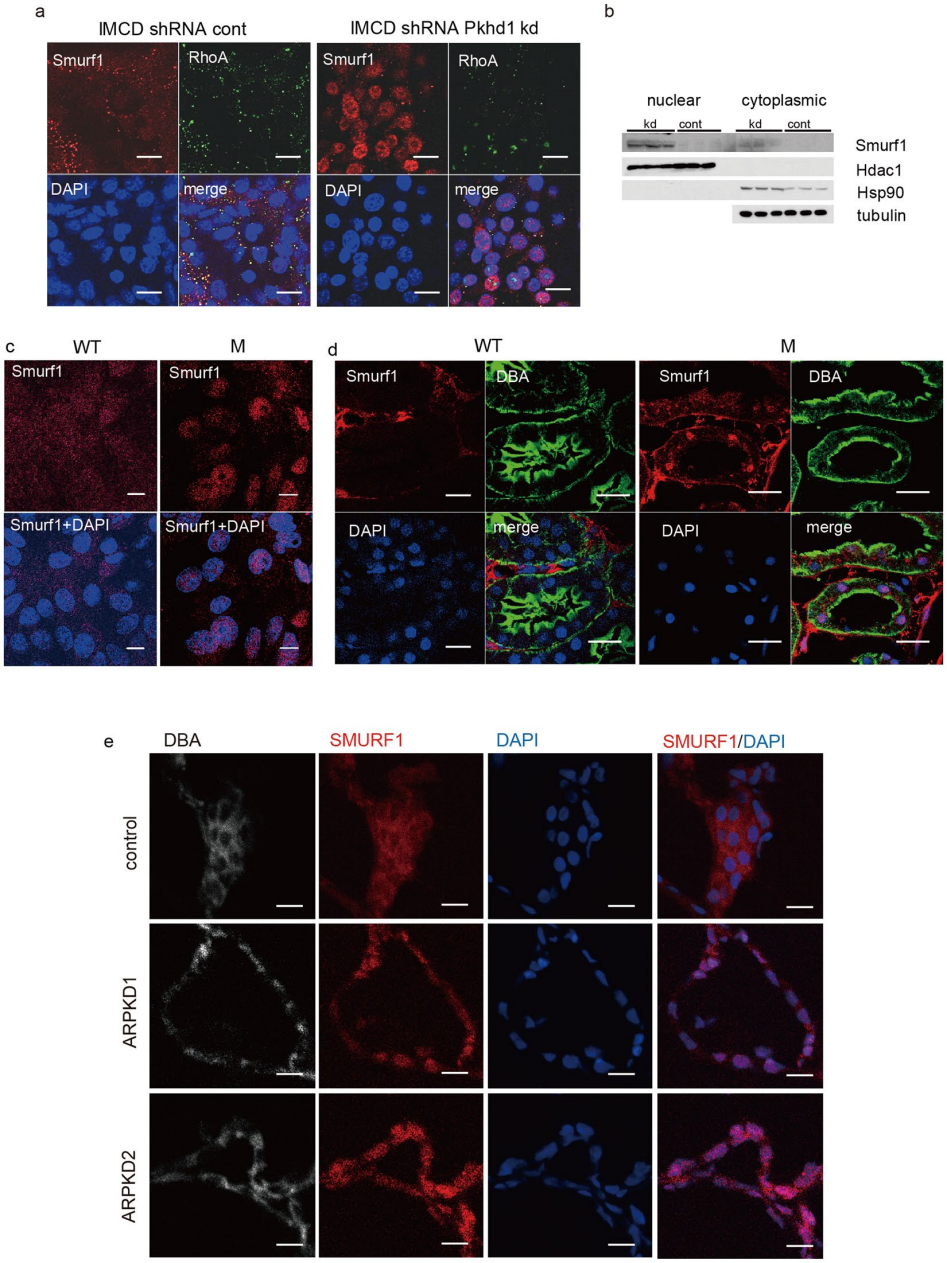
Smurf 1 is mis-localized in *Pkhd1* mutants. (**a**) Immunolocalization of endogenous Smurf1 (red) and RhoA (green) in IMCD cells with stable expression of control shRNA vector and *Pkhd1* shRNA. Smurf1 and RhoA co-localize in a subset of small vesicles in control cells with Smurf1 undetectable in the nucleus. In *Pkhd1*-silenced cells, Smurf1 localizes predominantly to the nucleus and is minimally detected in vesicles. Nuclei were stained with DAPI (blue). Scale bar, 10 μm. (**b**) Nuclear and cytoplasmic fractions of shRNA vector control IMCD cells (cont) and sh*Pkhd1* IMCD cells (kd) were immunoblotted as shown. Hdac1 and Hsp90 were used to show adequate separation of nuclear and cytoplasmic fractions, respectively. Tubulin was used as a loading control. These results are consistent with the IF data in panel “a” and show significant enrichment of Smurf1 in the nuclear fraction of IMCD cells with shRNA-reduced *Pkhd1* expression. The full-length blots of these cropped images are included in Supplementary Fig. S8. (**c**) Sub-cellular localization of Smurf1 (red) in primary cultures of collecting duct cells of wild type (WT) and *Pkhd1* ^del3-4/del3-4^ (M) mouse kidneys. Nuclei are stained with DAPI. Scale bars, 10 μm. (**d**) Wild type (WT) and *Pkhd1* ^del3-4/del3-4^ mouse kidneys stained for Smurf1 (red), DBA (green) and DAPI (blue). Scale bars, 20 μm. (**e**) Kidney specimens from human control (control) and two different ARPKD donors (ARPKD1, ARPKD2) stained for SMURF1 (red), DBA (grey), and DAPI (blue). Scale bars, 20μm.

Since Smurf1 is a major regulator of RhoA degradation and RhoA levels were increased while RhoA ubiquitination was reduced in mutant cells, we concluded that Smurf1 activity was likely altered in cells lacking *Pkhd1*. This prompted us to examine how changes in FPC function might affect its sub-cellular localization. In IMCD cells, Smurf1 was localized to small cytoplasmic vesicles in control cells but predominantly in the nuclei of *Pkhd1* shRNA-silenced cells (Fig. 2a,b). A similar pattern was observed in cultured primary collecting duct cells of wild type mice and *Pkhd1* ^*del3-4/del3-4*^ mutant mice (Fig. 2c). In studies of tissue, Smurf1 was barely detectable in WT kidneys of mouse and human but a prominent nuclear pattern was observed in the kidneys of both *Pkhd1* ^*del3-4/del3-4*^ mutants and human ARPKD specimens (Fig. 2d,e).

**Figure 3:**
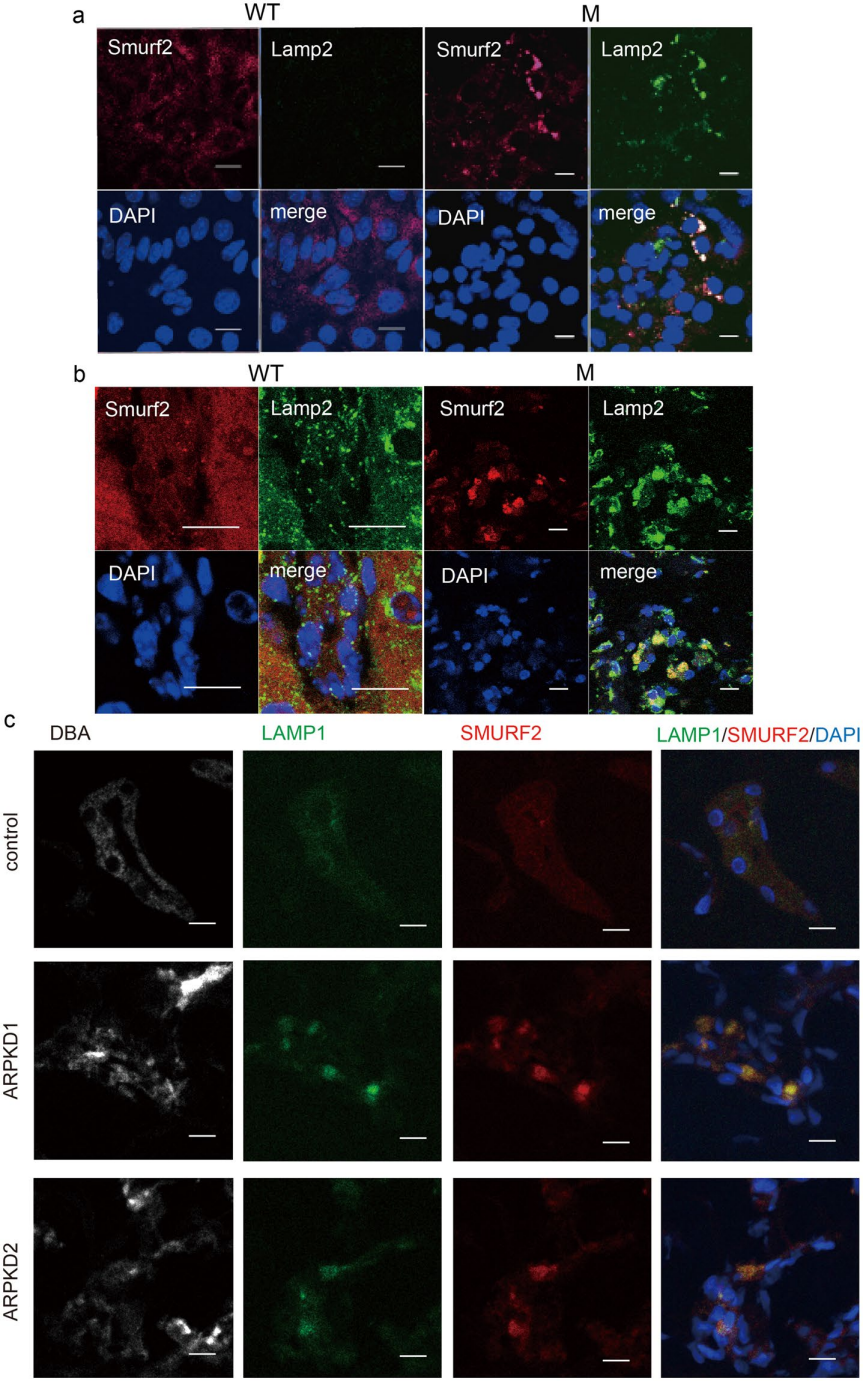
Smurf2 is mis-localized in *Pkhd1* mutants. (**a**) Sub-cellular localization of Smurf2 (red), Lamp2 (green) and DAPI (blue) in primary cultures of collecting duct cells of wild type (WT) and *Pkhd1* ^del3-4/del3-4^ (M) mouse kidneys. Nuclei are stained with DAPI. Scale bars, 10 μm. (**b**) Wild type (WT) and *Pkhd1* ^del3-4/del3-4^ mouse kidneys stained for Smurf2 (red), Lamp2 (green), and DAPI (blue). Scale bars, 10μm. (**c**) Kidney specimens from human control (control) and two different ARPKD donors (ARPKD1, ARPKD2) stained for DBA (grey), LAMP1 (green), SMURF2 (red) and DAPI (blue). Scale bars, 20 μm.

Smurf2 is a homologue of Smurf1 and linked to regulation of TGF-β signaling. Given that our studies show altered Smurf1 activity in *Pkhd1* mutant specimens and enhanced TGF-β signaling has been implicated as a cause of fibrosis in ARPKD tissues, we examined Smurf2 patterns of expression in cells and tissues with altered FPC activity. In control cultured collecting duct cells and in WT mouse and human kidney specimens, Smurf2 was found primarily in small vesicles but in the mutant cultured cells and ARPKD tissues it was localized to large vacuole-like structures that also stained for the lysosomal marker Lamp2 (Fig. 3). These data suggest that Smurf2 becomes trapped in large lysosomes in *Pkhd1* mutants.

Taken together, the data suggest that disruption of *Pkhd1* function results in dysregulated trafficking of both ubiqu itin E3 ligases, Smurf1 and Smurf2.

### PCK rat kidney has altered sub-cellular localization of Nedd4-2 and enhanced ENaC expression

**Figure 4:**
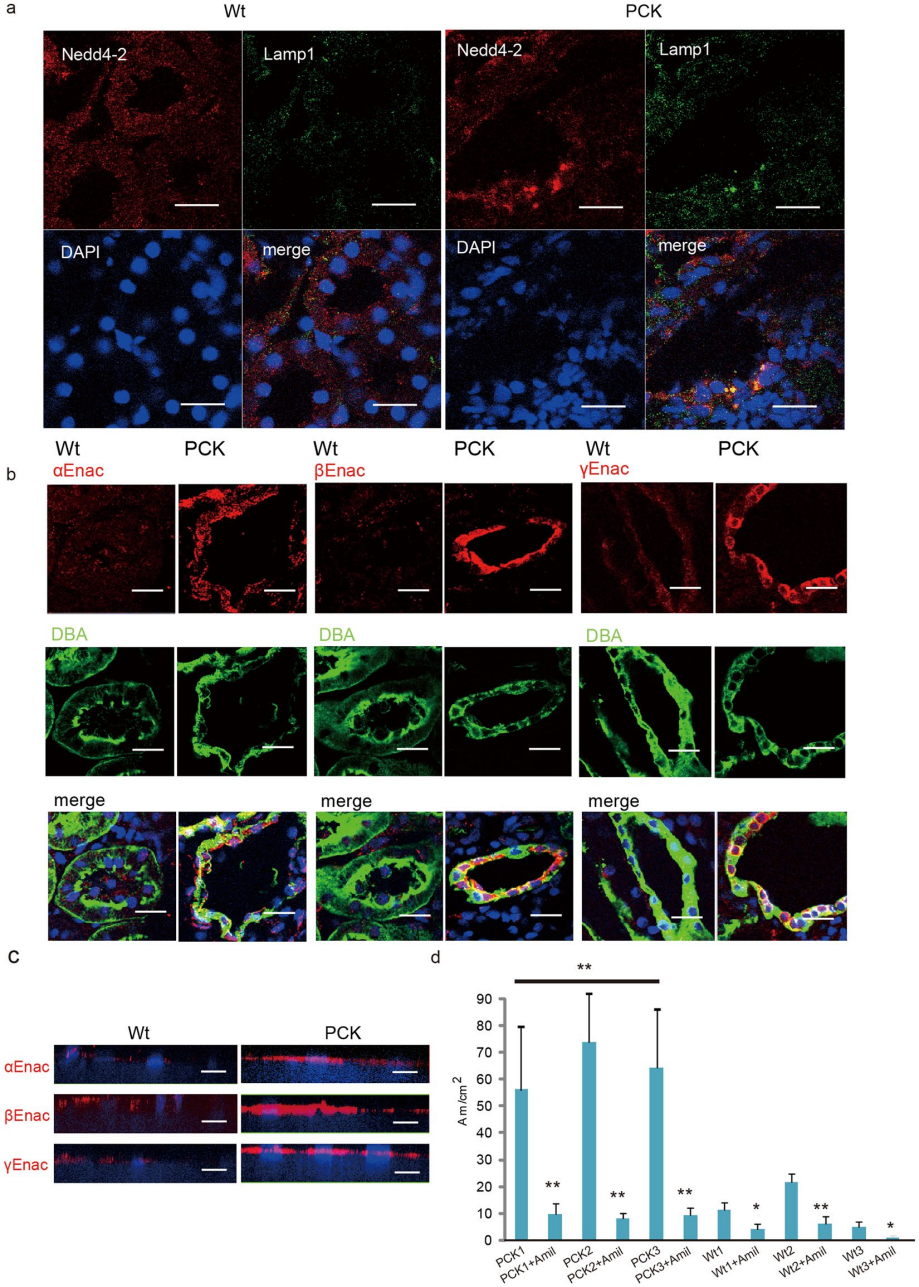
Nedd4-2 mis-localization in PCK kidney is associated with increased apical ENaC and enhanced sodium reabsorption. (**a**) Kidneys from 12-week old strain control (Crj:CD/SD) (Wt); left) and PCK (right) rats immunostained for Nedd4-2 (red), Lamp1 (green) and DAPI (blue). Similar patterns were observed in sections from three different mutants, and abnormalities were restricted to collecting ducts. Scale bars, 20 μm. (**b**) Kidneys from 12-week old strain control (Wt) and PCK rats immunostained for each of the three subunits of ENaC (red), DBA (green) and DAPI (blue). Scale bars, 20 μm. (**c**) Primary CD cells from 12-week old strain control and PCK rats were isolated and cultured on Transwell filters and then immunostained for each of the three ENaC subunits (red) and DAPI (blue). Scale bars, 10 μm. (**d**) Open-circuit measurements of primary CD cells from three 12-week old strain control rats (Wt1, Wt2 and Wt3) and three PCK rats (PCK1, PCK2, PCK3) cultured on Transwell filters either in the absence or presence (“+Amil) of 50 μM amiloride in the apical side. Measurements were collected from at least 10 independent wells per animal. *P < 0.05, ** P < 0.01.

Smurf1 and Smurf2 are members of the C2-WWW-HECT domain E3 family of ubiquitin ligases. The observation that *Pkhd1* dysfunction alters the localization and function of two different E3 ligases prompted us to consider whether *Pkhd1* mutation might affect any others. In the CD of the kidney, the E3 ligase Nedd4-2 is a major regulator of ENaC activity by promoting endocytosis and degradation of the ENaC complex^35, 36^. Enhanced ENaC degradation limits sodium reabsorption in the CD while a reduction in Nedd4-2 function results in enhanced sodium re-uptake. Given that hypertension is nearly a universal feature of human ARPKD and attributed to increased distal sodium reabsorption by some investigators^3, 4^, we examined Nedd4-2 localization in the PCK rat kidney. This orthologous model develops hypertension by four months of age^37^. In control kidneys, Nedd4-2 co-localizes with the lysosomal marker Lamp1 in small vesicles distributed throughout the cytoplasm of CD cells (Fig. 4a). In PCK kidneys, Nedd4-2 and Lamp1 are co-localized in large vacuole-like structures (Fig. 4a) similar to what was seen for Smurf2 (Fig. 3b). The same localization changes in NEDD4-2 were observed in ARPKD patient tissues (Supplementary Fig. S3). Consistent with Nedd4-2’s role as a major regulator of ENaC, we found increased levels of the three ENaC subunits in the apical membrane of PCK rat kidneys (Fig. 4b) and in the apical membrane of cultured PCK CD cells (Fig. 4c). Finally, we confirmed that these differences had functional significance by comparing rates of sodium reabsorption of cultured CD cells isolated from three different pairs of wild type Crj:CD and PCK rats. Sodium transport by the PCK CD cells was 3-7X higher than that of controls, and this activity was completely blocked by amiloride, a known inhibitor of ENaC (Fig. 4d).

### Ndfip2, a Nedd4 family interacting protein, co-precipitates with FPC from intracellular vesicles

**Figure 5:**
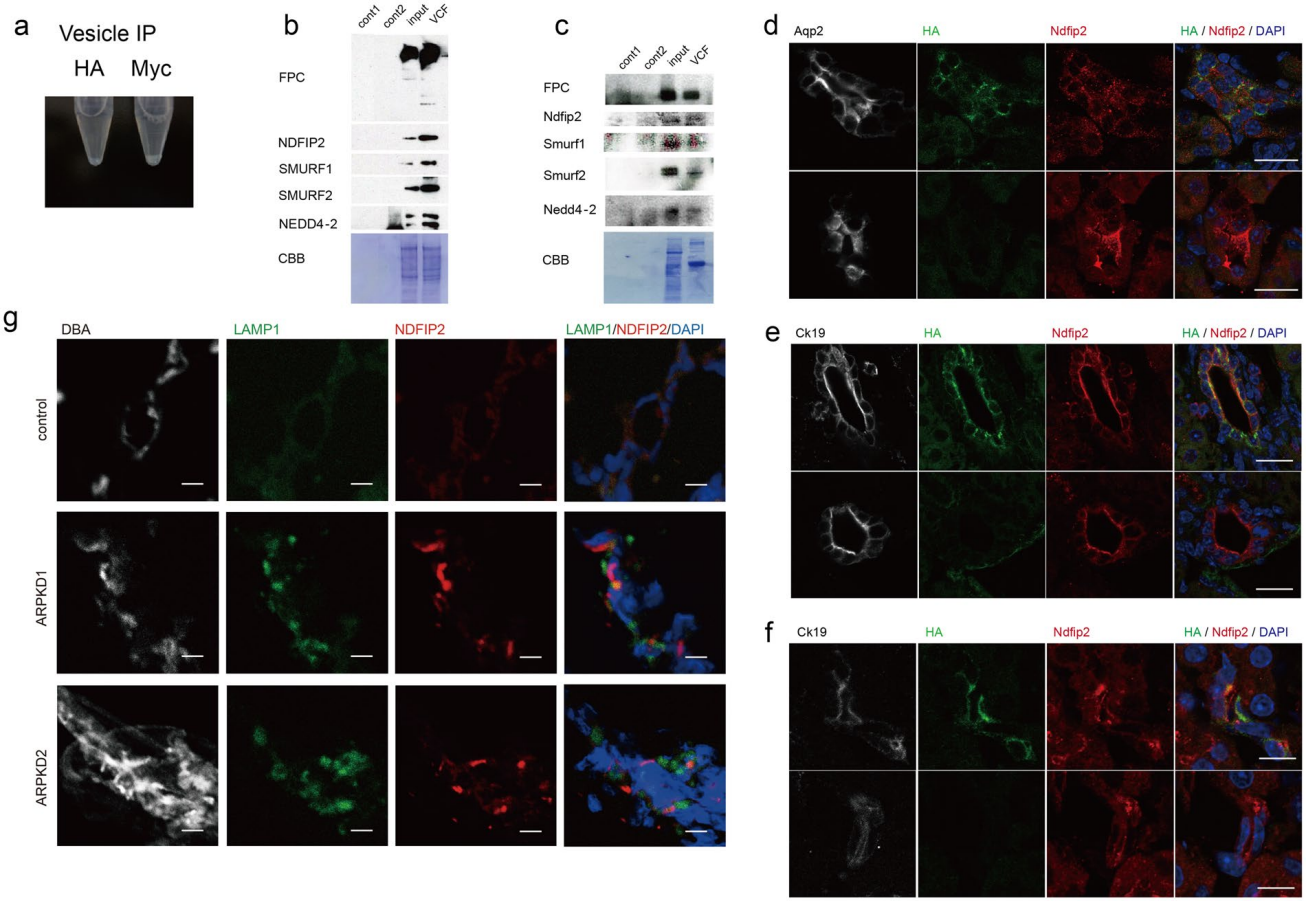
FPC positive vesicles contain NEDD4 interacting protein NDFIP2. (**a**) Microtube containing pellet of vesicles isolated from HEK cells expressing *PKHD1-Myc* using either anti-HA or anti-Myc coupled beads. A small pellet is visible in the anti-Myc sample but not in the anti-HA negative control. (**b**) Immunblot of vesicles isolated using either anti-Myc (lanes 1,4) or anti-FLAG (lane 2) coupled beads from HEK cells with either vector control (lanes 1,2) or expressing *PKHD1-Myc* (lane 4) and analyzed with antibodies against Myc, NDFIP2, SMURF1, SMURF2 and NEDD4-2. Lane 3 contains 20 μg of total lysate of the *PKHD1-Myc* sample. Total protein quantity was accessed by Coomassie brilliant blue staining (CBB). (**c**) Immunblot of vesicles isolated using either anti-HA (lanes 1, 4) or anti-FLAG (lane 2) coupled beads from fresh kidneys of wild type (lanes 1, 2) or *Pkhd1* ^*HA-Flox*^ mice (lane 4) and probed with antibodies against HA, Ndfip2, Smurf1, Smurf2 and Nedd4-2. Lane 3 contains 20 μg of total lysate of the *Pkhd1* ^*HA-Flox*^ sample. Total protein quantity was assessed by Coomassie brilliant blue staining (CBB). Anti-HA detects full length Fpc. The full-length blots of cropped images in b and c are included in Supplementary Fig. S9. (**d–f**) Confocal analysis of kidney (**d**), liver (**e**), and pancreas (**f**) from *Pkhd1* ^*HA-Flox*^ (top panels in each) or wild type mice (bottom panels in each) for Fpc (HA) (green), Ndfip2 (red), DAPI (blue) and markers for collecting duct (aquaporin 2, Aqp2) or epithelial cells (cytokeratin 19, Ck19). Merged images are on far right. Scale bars, 20 μm in “d” and “e” and 10 μm in “f”. (**g**) Kidney specimens from human control (control) and two different ARPKD donors (ARPKD1, ARPKD2) stained for DBA (grey), LAMP1 (green), NDFIP2 (red) and DAPI (blue). Scale bars, 10 μm.

The effects of *Pkhd1* mutation on the subcellular patterns of localization for Smurf1, Smurf2 and Nedd4-2 prompted us to test for a direct interaction with recombinant FPC. Unable to co-precipitate the proteins reproducibly under standard conditions, we isolated vesicles containing FPC from HEK293 cells with stable expression of recombinant *PKHD1* and re-tested for co-precipitation^18^ (Fig. 5a). Using this approach, we detected all three co-precipitating with FPC (Fig. 5b). Because FPC co-precipitated with multiple members of the C2-WWW-HECT domain E3 family, we tested for co-precipitation of NDFIP2, a ubiquitin ligase interacting protein implicated in regulating the trafficking and function of the NEDD4 ubiquitin ligase family^21^ ^22^. NDFIP2 also was present in the vesicles (Fig. 5b). These *in vitro* western blotting data were also confirmed *in vivo* by western blotting after vesicle IP using kidneys from HA tagged *Pkhd1* exon 67 knock-in mice (*Pkhd1* ^*HA-Flox*^) (Fig. 5c).

We next determined the sub-cellular pattern of FPC and Ndfip2 co-expression in normal and ARPKD mutant specimens. In HEK cells with stable expression of Myc-tagged recombinant *PKHD1*, we found endogenous NDFIP2 co-localized with FPC in a small subset of vesicles in super-resolution microscopy images (Supplementary Fig. S4a). In tissues of *Pkhd1* ^*HA-Flox*^ mice, FPC partially co-localized with Ndfip2 in collecting duct, biliary ducts and pancreatic ducts (Fig. 5d,e,f). Both proteins had a prominent apical pattern in the bile and pancreatic ducts where they partially co-localized with cytokeratin 19, an epithelial cell marker, whereas the pattern was more diffuse in the kidney. In mutant specimens, Ndfip2 was primarily localized to large, Lamp1-negative vacuole-like vesicles in primary collecting duct cells cultured from *Pkhd1*-mutant mice (Supplementary Fig. S4b) and in human ARPKD kidney samples (Fig. 5g). Collectively, these data suggest that disruption of *PKHD 1* function affects NDFIP2 trafficking.

### PKHD1 deficiency results in altered endocytosis and global membrane processing defects

**Figure 6:**
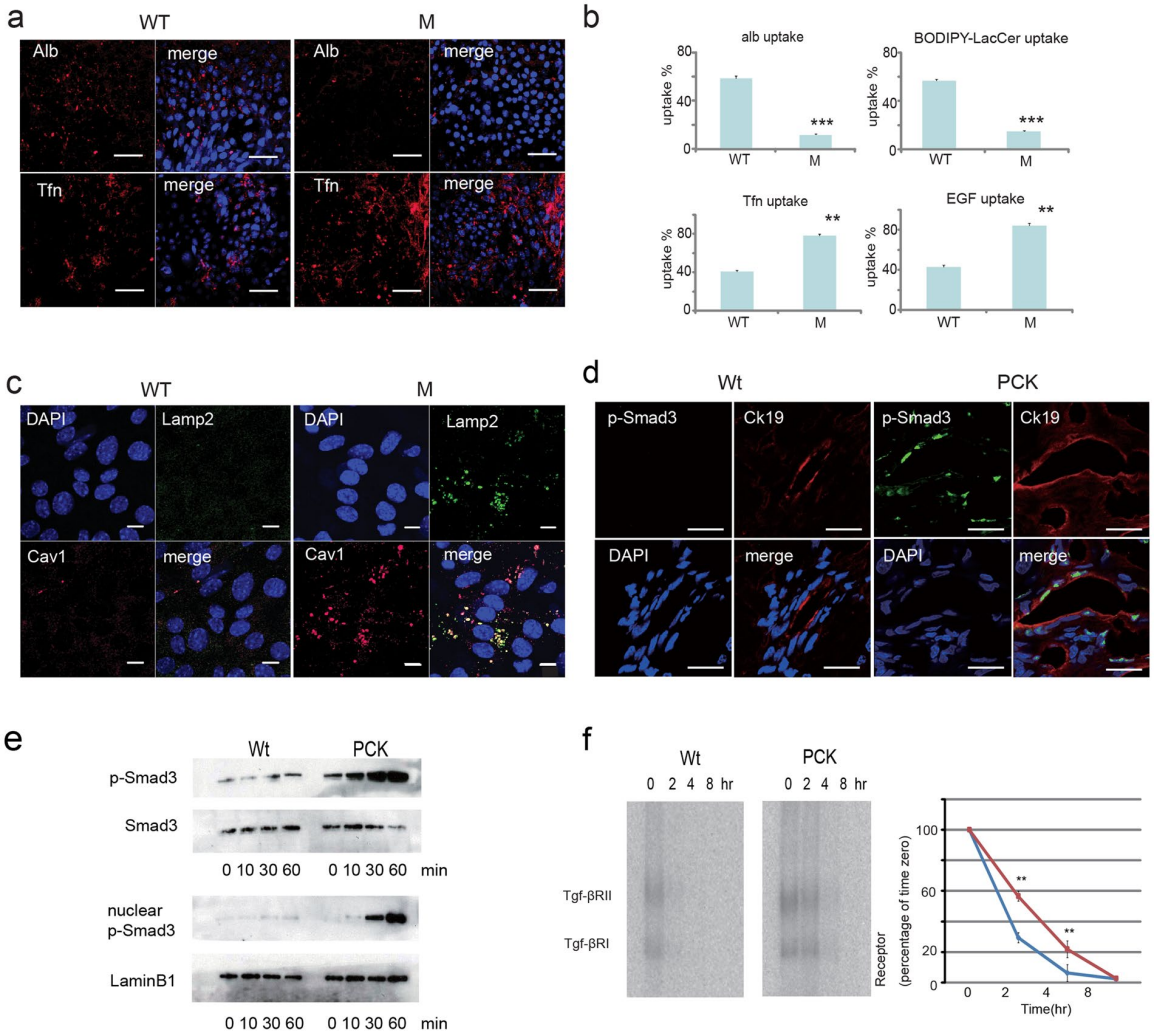
*Pkhd1* ^del3-4/del3-4^ collecting duct cells have abnormal endocytosis and membrane protein partition defects. (**a**) Endocytosis assays in primary confluent cultures of collecting duct cells isolated from control (WT) and *Pkhd1* ^del3-4/del3-4^ mouse kidneys (M) using fluorescently-labeled markers for the caveolar [albumin (Alb, red)] and clathrin [transferrin (Tfn, red)] endocytic pathways. Nuclei were stained with DAPI. Scale bars, 50 μm. (**b**) Quantitation of results for internalization assays using fluorescent markers for the clathrin (Tfn, EGF) and caveolar (alb, LacCer) endocytic pathways in confluent primary cultures of collecting duct cells from control (WT) and *Pkhd1* ^del3-4/del3-4^ (M) mice. Data were obtained using conventional fluorescence and are expressed as percent of uptake. Data represent quantification of at least 100 cells from each of three independent experiments. ** P < 0.01, *** P < 0.001. (**c**) Primary confluent cultures of collecting duct cells isolated from control (WT) and *Pkhd1* ^del3-4/del3-4^ mouse kidneys (M) were stained for markers of lysosomes (Lamp2, green) and caveolin-1 containing lipid-raft rich vesicles [Cav1 (red)] and DAPI (blue). The merged image shows co-localization of caveolin-1 with Lamp2 in large, vacuole-like lysosomes in the mutant cells. Scale bars, 10 μm. (**d**) Liver specimens of 12-week old Crj:CD/SD (Wt) (control) and PCK rats were stained for phospho-Smad3 (p-Smad 3, red), cytokeratin 19 (Ck19, red) and DAPI (blue). Scale bars, 20 μm.(**e**) Cell lysates of Crj:CD/SD (Wt) (control) and PCK mutant cholangiocyte cell lines treated with TGF-β for the indicated times and fractionated into cytoplasmic (top two panels) and nuclear (bottom two panels) fractions. Immunoblots were probed for phospho-Smad3 (p-Smad3) and total Smad3. Total Smad3 and LaminB1 were used as loading controls for the cytoplasmic and nuclear fractions, respectively. (**f**) Tgf-β receptor degradation assay in rat cholangiocyte cell lines. On the left, autoradiographs of a representative experiment for Crj:CD/SD (Wt) (control) and PCK mutant cholangiocyte cell lines treated with [125I]-labeled human TGF-β and then analyzed for levels of labeled Tgf-β receptor subunits at the times indicated. Three separate experiments were carried out, quantified by phosphorimaging and graphed as receptor quantity (% of time 0) vs. time (graph on right). Each point represents the mean ± SD. The results for wild type are in blue and mutant in red. ** P < 0.01. The full-length blots of cropped images in e and f are included in Supplementary Fig. S10.

Our data suggest that disruption of *PKHD1* activity results in altered trafficking of multiple proteins. A recent systems survey of endocytosis by multiparametric image analysis recently identified *PKHD1* as one of 4609 genes whose function was required for normal endocytic processes^38^. Taken together, these observations suggest that changes in *Pkhd1* function might affect endosomal trafficking, so we next examined rates of endosomal internalization using functional assays. We first analyzed lipid raft caveolar-dependent endocytosis by quantifying uptake of fluorescently-labeled albumin and Lactosylceramide (BODIPY-LacCer) and found that both were significantly reduced in *Pkhd1* ^*del3-4/del3-4*^ primary CD cells and in IMCD cells with siRNA-reduced *Pkhd1* expression (Fig. 6a,b; Supplementary Fig. S5a,b). Conversely, MDCK cells with increased expression of *PKHD1* had significantly greater uptake of these markers (Supplementary Fig. S5c,d). We then compared rates of clathrin-dependent endocytosis using labeled transferrin (Tfn) and Epidermal Growth Factor (EGF) as markers and found opposite results. Uptake was markedly increased in cells with reduced *Pkhd1* function but reduced in those with increased *PKHD1* expression (Fig. 6a,b; Supplementary Fig. S5a–d).

SMURF2 is known to associate with caveolin-rich lipid rafts^39^. Given the effects of reduced *Pkhd1* activity on SMURF2 localization and function and the generalized trafficking defects, we examined the pattern of expression of the lipid raft vesicle marker caveolin-1 in primary CD cells isolated from wild type control and *Pkhd1* ^*del3-4/del3-4*^ mutant mice. In cells isolated from the mutant mice, caveolin-1 positive endosomes that result from lipid raft-caveolar internalization were more numerous and larger, appearing as vacuole-like structures also positive for Lamp-2 (Fig. 6c).

### *Pkhd1*-mutant cholangiocytes have enhanced TGF-β signaling due in part to reduced TGF-β receptor degradation

The regulation of TGF-β activity is a complex process that involves many components. One important aspect of this is how the heterodimeric receptor is processed after ligand binding. Clathrin-dependent internalization into EEA1-positive endosomes promotes TGF-β signaling while internalization via the lipid raft-caveolar pathway is required for rapid receptor turnover and signal termination^39^. SMURF2 and Smad7 play a key role in this latter process. Smad7 functions as an adapter to recruit SMURF2 to the membrane where the complex binds to activated TGF-β receptor and targets the TGF-β receptor for degradation^6, 40, 41^. Our studies suggest that decreases in *Pkhd1* function may result in increased TGF-β activity by disrupting SMURF2-dependent degradation pathways.

We therefore compared the TGF-β signaling system in wild type and *Pkhd1*-silenced IMCD cells and wild type and *Pkhd1* mutant cholangiocytes. As expected, we found pSmad3, a marker for TGF-β activation, in the nuclei of cholangiocytes lining bile ducts of PCK mutants (Fig. 6d). Consistent with this observation, treatment of the PCK cholangiocyte cell line with 100 pM recombinant TGF-β resulted in robust phosphorylation and nuclear translocation of Smad3 compared to control (Fig. 6e). A TGF-β receptor degradation assay in the same cell lines revealed that TGF-β receptor degradation was significantly compromised in PCK cholangiocytes compared with controls (Fig. 6f). *Pkhd1*-silenced IMCD cells also had delayed TGF-β receptor degradation compared to control cells (Supplementary Fig. S6).

## Discussion

ARPKD is a significant human genetic disease that affects the kidney and liver with high morbidity and mortality. Despite identification of *PKHD1*, the mutant gene responsible for the disorder, and the availability of orthologous rodent models, little is known about the pathogenesis of the disease. Affected individuals typically develop hypertension, renal failure and portal tract fibrosis at a young age but the cellular defects responsible for these clinical features have only been partially characterized.

**Figure 7:**
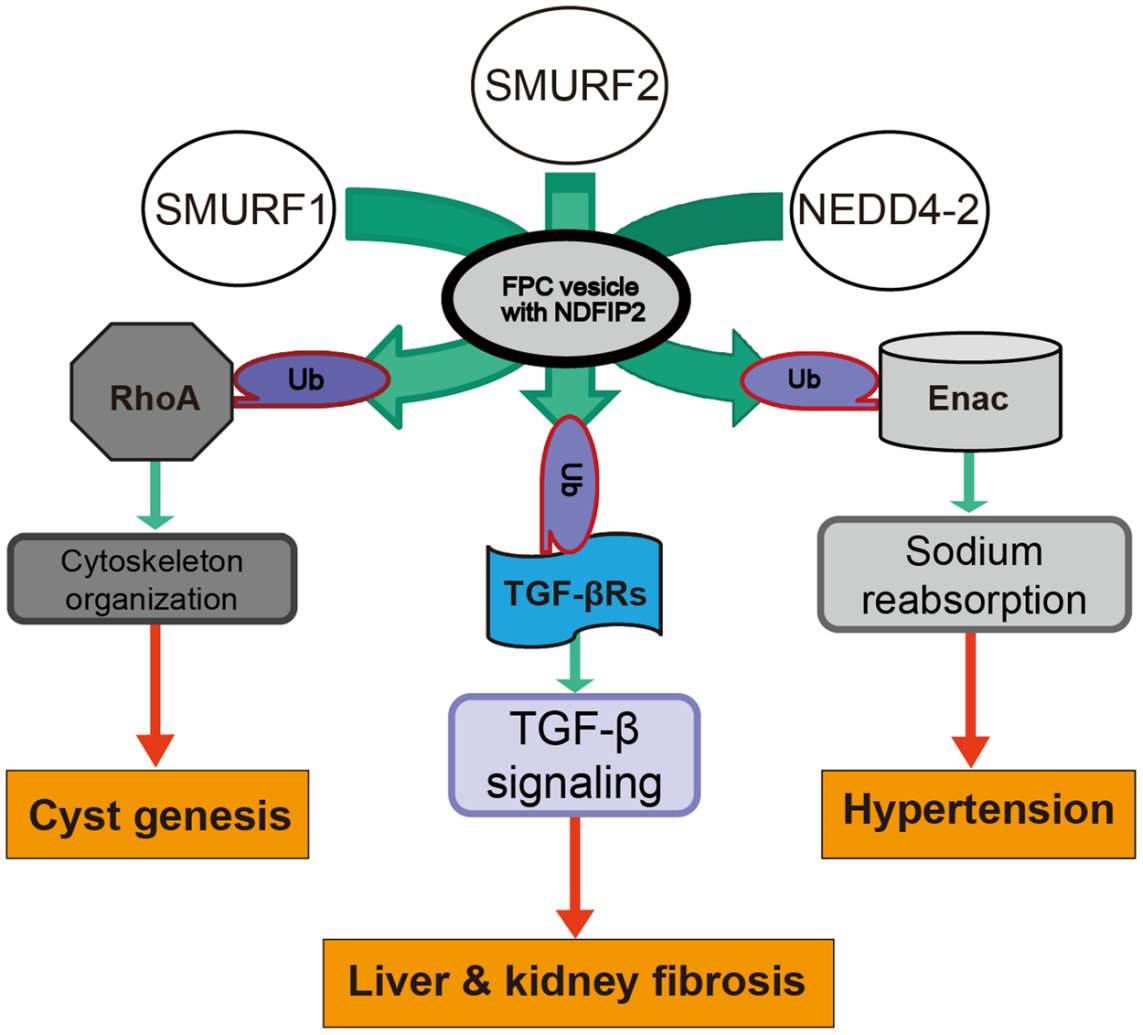
Schematic presentation of the functional properties of FPC. FPC exerts its effects on the cytoskeleton and cell-cell junctions by indirectly regulating the activity of RhoA via the E3 family of ligases. FPC helps maintain balanced TGF-β signaling by regulating the targeting of SMURF1 and 2 to TGF-β receptors after ligand binding. FPC indirectly affects sodium balance by regulating the activity of NEDD4-2 in the CD and thereby affecting degradation of ENaC. These all functions are conducted through NDFIP2 in FPC positive vesicle.

In the current work, we have used multiple complementary approaches to identify a novel link between the *PKHD1/Pkhd1* gene product, FPC, and the C2-WWW HECT domain E3 family of ubiquitin ligases. We show that FPC positive vesicles contain NDFIP2, a NEDD4 ubiquitin ligase family regulator and that reducing the activity of FPC results in altered sub-cellular trafficking of at least three different E3 family members SMURF1, SMURF2, and NEDD4-2. This in turn results in reduced ubiquitination of their targets and important functional consequences that are likely key steps in the pathophysiology of the disease (Fig. 7).

SMURF1 is an important regulator of the activity of the RhoA. We found that reducing FPC activity results in reduced levels of ubiquitinated RhoA, a major regulator of F-actin structure and an important determinant of cellular morphology, and significant changes in the actin cytoskeleton. Our findings are consistent with what was previously reported for *Pkhd1*-silenced IMCD cells^28^, and we now provide a mechanistic explanation for these observations.

E3 ubiquitin ligases also play an important role in regulating TGF-β signaling. TGF-β signaling is modulated by two distinct endocytic pathways. Positive TGF-β signals are conveyed to the nucleus by EEA1 positive endosomes that begin with clathrin-dependent internalization, whereas activated TGF-β receptors are down-regulated by caveolin positive endosomes that result from lipid-raft/caveolar internalization^39^. SMURF2 plays a major role in this latter step^42^. We found that multiple components which regulate this signaling system were altered in *Pkhd1*-deficient cells. Epithelial cells with reduced *Pkhd1* function had enhanced clathrin-mediated endocytosis, reduced raft-mediated endocytosis and decreased SMURF activity. Consistent with these findings, we found increased TGF-β activation in *Pkhd1-*deficient cells. We speculate that these mechanisms may explain the increased TGF-β signaling previously reported in *Pkhd1* livers and contribute to the hepatic fibrosis that is a universal feature of ARPKD (Fig. 7). Our findings provide a novel explanation for how this important clinical problem develops. These results also may help to explain the fibrosis seen in the cystic kidney since TGF-β1 plays a key role in the onset of renal fibrosis and progression of chronic renal disease.

These data have potential therapeutic implications. The angiotensin II type 1 receptor signaling system is an important modulator of TGF-β activity, and inhibition of this pathway with angiotensin receptor blockers (ARBs) is effective in reducing TGF-β signaling in other disease states. ARBs prevented aortic aneurysm in a murine model of Marfan syndrome and inhibited aortic-root dilation in humans with the same condition^43, 44^. It will be interesting to see if ARB therapy can delay biliary and renal fibrocystic disease progression in pre-clinical rodent models.

Hypertension is another nearly universal clinical feature in ARPKD. The mechanisms underlying this clinical issue are largely unknown but thought in part to be due to enhanced sodium reabsorption in the collecting duct by the epithelial Na (+) channel (ENaC)^3, 45, 46^. Our findings provide a possible explanation for this phenomenon (Fig. 7). We show that PCK mutants have altered cellular localization of Nedd4-2, increased levels of apical ENaC and increased sodium reabsorption that is blocked by amiloride. These data support the hypothesis that chronic enhanced sodium reabsorption is an important pathogenic contributor to hypertension in this disorder. Our findings also suggest that sodium-channel blockers such as amiloride might be particularly effective as an anti-hypertensive agent in the clinical management of affected individuals.

The effects of *Pkhd1* on SMURF and RhoA activity may also contribute to cyst formation by other mechanisms. Recent studies suggest that abnormalities in planar cell polarity (PCP) may play a pathogenic role in causing polycystic kidney disease [reviewed in ref. 47]. PCP refers to the phenomenon whereby cells coordinate their orientation with respect to the plane of a tissue. This is especially important during kidney development when tubular epithelial cells are rapidly dividing to produce elongated tubular structures. Oriented cell division is one mechanism by which PCP can be established, and defects in this process have been reported in kidneys of the PCK rat, an orthologous rodent model of ARPKD^48^. The SMURF ubiquitin ligases have recently been reported to play an essential role in regulating PCP by targeting the core PCP protein Prickle1 for ubiquitin-mediated degradation^49^. Given the pronounced effects that changes in *Pkhd1* activity have on SMURF localization and function, we hypothesize that this may be an important mechanism linking *PKHD1* to PCP pathways.

In this study, we restricted our analysis to just three members of the C2-WWW-HECT domain E3 family of ligases and a very limited set of effector pathways. Our results suggest that disruption of *Pkhd1* function is likely to alter the activity of numerous other signaling pathways. It is reasonable to suspect that FPC may alter the activity of other E3 family proteins and other ubiquitination targets. We also found global effects on endocytosis, with enhanced activity of clathrin-dependent pathways and the opposite for lipid-raft caveolar-based internalization. Numerous other receptors signal through these systems so it is likely that their activity also may be abnormally regulated in *Pkhd1* mutants. If this model is correct, it will complicate our efforts to identify a principal function for FPC or a single pathway suitable for therapeutic targeting. As a parenthetical aside, so will the organ-specific transcriptional complexity of *PKHD1/Pkhd1*.

The mechanism by which *Pkhd1* inactivation causes these effects is incompletely understood. Our results suggest that FPC may have direct effects on E3 ligase function by altering the latter’s localization within the cell. The altered signaling that results may additionally affect cell trafficking, further contributing to the observed abnormalities. This model is consistent with findings of two independent genome-wide siRNA screens that investigated clathrin- and caveolae/raft mediated endocytic pathways^50^. Pelkmans *et al*. found that silencing of multiple kinases caused bidirectional changes in endocytosis consistent with what we observed. Collinet^38^ determined that signaling pathways such as TGF-β, Wnt, and others also regulate the endocytic system. It is interesting to note that *Pkhd1* was identified in the same screen as one of the new genes involved in regulating endocytosis, though the pattern in HeLa cells differed from what we observed.

Finally, much has been made of the ciliary localization of many cystoproteins, including FPC^5, 13–17, 51^. In the current study, we did not examine potential mechanistic links between *Pkhd1*-related functional defects in the E3 ligase family and the primary cilium. There is, however, a growing appreciation of the inter-dependence of intracellular and ciliary trafficking pathways for cellular function. For example, Rab8, Rabaptin-5 and IFT-20 have all been shown to play important roles in both^52–54^. Given the complex coordination that is required to organize cell structures in three dimensions, it is not surprising that there should be cross-talk between the various cellular trafficking and signaling pathways. Our studies suggest these processes are dysregulated in ARPKD.

## Methods

### Rodents

The *Pkhd1* ^*del3-4/del3-4*^ mouse was described previously^29^. *PCK/CrljCrl-Pkhd1pck/pck* (PCK) and Crj:CD (control) rats were purchased from Charles River Japan (Sagamihara, Japan), and a colony of PCK rats was maintained at the Laboratory Animal Institute of Kanazawa University School of Medicine, Kanazawa, Japan. All rat experiments were conducted in accordance with the Guidelines for the Care and Use of Laboratory Animals at Takara-machi Campus of Kanazawa University. Mouse studies were performed using protocols approved by the University Animal Care and Use Committee of Osaka University (approval number: DOI 25-046-3), Johns Hopkins University, and the NIH, and mice were kept and cared in pathogen-free animal facilities accredited by the American Association for the Accreditation of Laboratory Animal Care and meet federal (NIH) guidelines for the humane and appropriate care of laboratory animals. The PCK rat model was substituted for the *Pkhd1* ^*del3-4/del3-4*^ mouse line during the study because the mouse model lost its renal cystic phenotype after backcrossing into the C57Bl6 strain to make a congenic strain.

### Generation of 3xHA tagged *Pkhd1* knock-in mouse lines

The *Pkhd1* ^*HA-Flox*^ mouse line will be described elsewhere (manuscript in review). Briefly, this line has exon 67 flanked by lox P sites and three copies of the HA tag “knocked-in” in-frame to the C-terminus of FPC in exon 67. In our previous *in vitro* studies, we had found that similar modification did not change the trafficking properties of FPC^18^ in MDCK cells. After establishing a heterozygous *Pkhd1* ^*HA-Flox*^ line, we excised the neo cassette by crossing with a Flp-expressing mouse.

### Antibodies and reagents

A complete list of antibodies, a lectin and vendors is presented in Supplementary Table S1. Anti-NDFIP2 and anti-ENaCs antibodies were previously described^55, 56^. The fluorescent probe-conjugated secondary antibodies used in immunostaining studies were purchased from Molecular probes/Invitrogen and were used at a dilution of 1:400. Other reagents included FITC-or TRITC-conjugated phalloidin (Sigma and Vector lab, respectively), Alexafluor 555-conjugated bovine serum albumin and transferrin (Tfn) (Molecular probes/Invitrogen), Texas red Epidermal Growth Factor (EGF) complex and BODIPY-C5 lactosylceramide (Molecular probes/Invitrogen).

### Cell lines

The *Pkhd1*-silenced and control IMCD cell lines [IMCD *Pkhd1* siRNA (e23) and IMCD control siRNA (h21)] and the *PKHD1*-expressing and control MDCK cell lines (MDCK cont1, MDCK cont2, MDCK PKHD1 Myc1, MDCK PKHD1 Myc2, MDCK PKHD1 EGFP) have been previously described^18, 28^. The cholangiocyte cell lines derived from the PCK and control Crj:CD/SD rats were kindly provided by Dr. LaRusso (Mayo Clinic)^33^. The mouse derived collecting duct cell line was previously described^57^.

All cell lines were cultured according to published protocols.

### Human kidney tissue

ARPKD patients and healthy control tissue was collected with informed consent. This research was approved by Institutional Review Board of University of Alabama at Birmingham (approval number: X980102001) and University of Sao Paulo (approval number: 621/04). All experiments related to human kidney tissues were performed in accordance with relevant guidelines and regulations of the two facilities. A description of the donors is presented in Supplementary Table S2.

### Immunoblotting/immunoprecipitation

Cells were incubated in lysis buffer [20 mM Na phosphate (pH 7.2), 150 mM NaCl, 1 mM EDTA, 10% glycerol, 1% Triton X-100 and protease inhibitor cocktail (Roche)], centrifuged at 14000 RPM for 10 min to clear debris, and then equal amounts of protein as measured by Bradford assay were boiled, separated by SDS-PAGE, and then electrotransferred to Immobilon-P (polyvinylidenedifluoride) membranes (Millipore). Western blots were incubated overnight with primary antibodies at 1:1000 dilution. ECL-enhanced chemiluminescence was used for secondary antibody detection (GE Healthcare Life Science). Immunoprecipitation studies were performed as described previously with slight modifications^58^. Uncropped immunoblots are presented in Supplementary Figs S7–11.

### Rho activation assay

Quantification of GTP-bound RhoA was conducted using Rho Activation Assay Biochem Kit (Cytoskeleton, Inc.) according to the manufacturer’s instructions. Each confluent cell line in a 10 cm dish was lysed in cell lysis buffer. Cell lysates containing equal amount of protein underwent pulldown assay using Rhoketin beads in the kit. The pulldown samples were immunoblotted by anti RhoA antibody (Supplementary Table S1).

### Isolation of vesicles and immunoprecipitation (IP) of vesicles from cultured cells and mouse kidney tissues

Vesicle IP was performed as previously described^59^. Isolation of vesicles from cultured cells and mouse kidney tissues were performed by using 10 ml of dissecting buffer [0.3 M sucrose, 25 mM imidazole, and 1 mM EDTA, pH7.2, proteinase inhibitor before use (Roche complete 11697498001)]. HEK cells collected from ten P150 dishes were used for each IP. Four mouse kidneys per single IP (3-month old, male) were homogenized using a gentleMACS Dissociator (Miltenyi Biotec GmbH) with program D1. The collected cells and homogenates of mouse kidneys were re-suspended using a 20 G needle and 20 ml syringe for 20 times while on ice. The final homogenates from cultured cells and mouse kidneys were centrifuged at 1000 g for 15 min for removal of nuclei, mitochondria, and any remaining large cellular fragments. After consecutive ultracentrifuges at 17,000 g for 30 min and at 200,000 g for 60 min at 4 °C, the suspended vesicles from culture cells were immunoprecipitated with anti-Myc or anti-HA coupled beads overnight, then centrifuged at 3000 rpm. The beads were washed repeatedly with dissecting buffer and after a final centrifugation, captured proteins were eluted with SDS sample buffer, separated by SDS-PAGE and then subject to immunoblot analysis.

### Immunofluorescence and confocal microscopy

For immunocytochemistry, cells were cultured on 12-mm Transwell filters (Corning) in DMEM 10% heat-inactivated FCS, 5 μg/ml blasticidin and 150 μg/ml hygromycin B for Flp-HEK or-MDCK cells or 1 mg/ml G410 for siRNA stable IMCD cells. Cell culture conditions and immunostaining methods were as previously described^18^.

Kidney and liver mouse sections were isolated from ~17-month old littermates (*Pkhd1* ^*del3-4/del3-4*^ and wild type mice), fixed in 4% paraformaldehyde and embedded in paraffin. For rat tissues, kidney and liver specimens were harvested from 12-week old animals and fixed in 4% PFA-PBS for two hr at 4 ^o^C. After fixation, cryoprotection was performed by incubating the tissues in 4, 10, 15, 20% sucrose in 0.1 M phosphate buffer (pH 7.4). The tissues were frozen in 2-pentane chilled on liquid nitrogen^60^. All tissue specimens were cut into 10 μm sections, incubated with primary antibody at 4 °C overnight and then secondary antibody at 37 °C for one hr. All studies were done using samples from a minimum of three rodents of each genotype with similar results obtained. Specimens were washed in PBS, placed in mounting medium with DAPI (Vector) and imaged using a Zeiss Axiovert 200 microscope with 510-Meta or 710-Nle or confocal module (Carl Zeiss) using a Plan-Apochromat 63x/1.4 or 100x/1.4 NA DIC lens with Immersol 518 F oil at room temperature. AxioVision or Zen software (Carl Zeiss) was used to reconstruct 3D images from 0.2 μm Z sections. The fluorescent intensity analyses (Supplementary Fig S2a–c) were performed by using Zen software (blue edition). For super-resolution images (Supplementary Fig S4a), a super-resolution structured illumination microscopy (SR-SIM), ELYRA S1 (Carl Zeiss) was used.

For human kidney tissues, we used the Kawamoto method^61^ and a new adhesive film (Cryofilm, Leica microsystems, Tokyo, Japan) for the preparation of fresh frozen sections from cystic kidney tissues since human cystic tissues otherwise easily detached from the glass slides. After several sections were cut, we attached the adhesive film to the exposed cutting surface of a sample. We made 10 µm of sections on the film and dried the sectioned samples for 30 min at room temperature. We fixed them in 4% PFA for 5 min then in 100% EtOH for 5 min. The samples were then three washes for 5 min/each in PBS and then immunostained.

### Isolation of rodent primary collecting duct cells

Kidneys from 6-month old, male WT and mutant mice and 4–5 month old, male Crj:CD/SD (control) and PCK rats were minced by razor in HEPES-Ringer buffer (118 mM NaCl, 16 mM HEPES, 5 mM glucose, 3.2 mM KCl, 2.5 mM CaCl_2_, 1.8 mM KH_2_PO_4_, Ph7.4, sterilized by filter) with 250 U/ml penicillin, 250 µg/ml streptomycin. In the case of mutant mice, we confirmed the cystic phenotype before the kidneys were minced. The tissue fragments were digested in HEPES digest solution containing 0.2% collagenase type II (Gibco/Invitrogen) and 0.2% hyaluronidase type IV (Sigma) in a CO_2_ incubator for at least two hr with intermittent dissociation by pipetting every 30 min. The digest solution was centrifuged at 150 g for five min and washed with HEPES buffer twice. The digested tissue was re-suspended in one ml of HEPES with antibiotics (per one kidney) and incubated in a 1.5 ml tube with biotin-conjugated DBA (Vector) coated Dynabeads (Dynal/Invitrogen) (50 μl of Dynabeads per one kidney) at room temperature for 30 min with rotation. After discarding the supernatant, the tube was placed in the magnet platform and the beads were washed with one ml of HEPES buffer twice. The primary cells were released from beads by using releasing buffer (DNase I) dissolved in primary culture medium (DMEM/F12 10%FBS, 50 U/ml penicillin, 50 μg/ml streptomycin, 5 μg/ml insulin, 5 nM dexamethasone, 5 ng/ml sodium selenite, 5 μg transferrin, 1 nM triiodothyronine, 10 ng/ml EGF). The primary cells were plated on a 24 well Transwell or 10 cm dishes and kept in primary culture medium. In every study, only first passage primary cells were used. We confirmed that we had isolated similar cell populations from each pair of mice by immunoblotting using two different aquaporin 2 (AQP2) antibodies. We determined that AQP2 expression levels in isolated cells from these different animals were the same. Each primary cell culture was isolated from a single mouse, and each set of studies was done a minimum of three times using independently-isolated cultures of cells.

### Endocytosis assay

The endocytosis assay was described previously^62^. The methods were slightly modified for kidney cells. Cells plated on 24 well plates Transwell (0.4 mm pore size) (Corning) were washed with HEPES-buffered MEM (10 mM HMEM) at room temperature and then incubated with 2 µM BODIPY-LacCer for 10 min at 37 °C in a 5% CO_2_ incubator to induce endocytosis. After incubation, the medium was replaced with ice-cold HMEM without glucose, and the culture dishes were transferred to a 10 °C bath. Fluorescent lipid present at the cell surface was removed by incubating the cells (six times, 10 min each) with 5% fatty acid free BSA in HMEM without glucose at 10 °C. For other experiments, cells were incubated with 7.5 mg/ml Alexa Fluor 594-labeled albumin, Tfn and EGF for 10 min at 37 °C. Excess fluorescent markers at the cell surface were removed by acid stripping (30 s at 10 °C with HMEM, pH 3.5). Confocal microscopy was performed using a Zeiss model 510 instrument and a 63× (1.4 NA) objective. Data represent quantification of at least 100 cells from at least three independent experiments using cells isolated from unique mice with Zen software program (Carl Zeiss).

### TGF-β receptor degradation assay

IMCD cells, mouse primary collecting duct cells and rat cholongiocyte cells were incubated with 100 pM [^125^I]TGF-β1 (Perkin Elmer) in KRH-0.5%BSA at 4 °C for 2 h and receptors were cross-linked to ligand with disuccinimidyl suberate (DSS, Thermo) as described previously^63^. Cell lysates were immunoprecipitated with anti-TGF-βRII antibodies, and receptors were visualized by SDS–polyacrylamide gel electrophoresis and autoradiography. Receptor levels were quantified using a phosphorimager (Fujifilm BAS-5000).

### Transepithelial electrophysiology

These experiments were performed according to previously described methods^45^. Rat collecting duct (CD) cells were isolated from 12-week old male rats on standard diets using methods similar to those described above for mouse CD primary cultures except the primary culture medium was DMEM-Ham’s F-12 medium supplemented with 10% FBS, 1.3 μg/l sodium selenite, 1.3 μg/l triiodothyronine, 5 mg/l insulin, 5 mg/l transferrin, 5 μM dexamethasone, 2.5 mM L-glutamine, 100 U/ml penicillin, and 100 μg/ml streptomycin. Rat primary CD cells were seeded onto 6.5-mm-diameter Costar Transwell filter supports (0.45-μm-diameter pore size polycarbonate filters) coated with diluted Matrigel solution at a high density at *day 0* (i.e., the seeding day). Open-circuit measurements of *R*te and *V*te using a Millipore Voltohmmeter were obtained on *day 2* after seeding with Ag-AgCl electrodes. Cells were harvested from three rats of each genotype and cultured in a minimum of 10 separate wells per rat. Transport studies were performed on a minimum of 10 wells for each primary cell culture. For ENaC inhibitor studies, *V*te was measured with apical application of 50 μM amiloride. During the measurement, *R*_te_ of these monolayers was 18–20 kΩ·cm^2^. The measurement was performed when *R*te and *V*te were stable. In the case of application of an inhibitor, open-circuit measurements were made after 5 min after the addition of amiloride. The current densities of these monolayers were calculated based on *R*te and *V*te.

### Statistical Analysis

Data were analyzed using ANOVA or Student’s *t*-test. ANOVA and Student’s *t*-test were performed using GraphPad Prism software (GraphPad Software, Inc.). A p value < 0.05 was considered to indicate statistical significance. The error bars indicate the standard deviation.

## Electronic supplementary material

Supplementary Information

Dataset 1
