# Supplementary material for: NEDD4-family E3 ligase dysfunction due to PKHD1/Pkhd1 defects suggests a mechanistic model for ARPKD pathobiology
Source: Sci Rep. 2017 Aug 10;7:7733. doi: 10.1038/s41598-017-08284-4 (PMC5552802; doi:10.1038/s41598-017-08284-4)
Supplement: Supplementary file 1 — Supplementary Information [file 41598_2017_8284_MOESM1_ESM.pdf]

NEDD4-family E3 ligase dysfunction due to *PKHD1/Pkhd1* defects suggests a mechanistic model for ARPKD pathobiology

Jun-ya Kaimori[1,2*], Chen-Chao Lin[3], Patricia Outeda[4], Miguel A Garcia-Gonzalez[5], Luis F Menezes[3], Erum A. Hartung[6] , Ao Li[7], Guanqing Wu[7], Hideaki Fujita[8], Yasunori Sato[9], Yasuni Nakanuma[10], Satoko Yamamoto[1], Naotsugu Ichimaru[2], Shiro Takahara[2], Yoshitaka Isaka[1], Terry Watnick[4], Luiz F. Onuchic[11], Lisa M. Guay-Woodford[12], Gregory G Germino[3,13*]

[1] Department of Advanced Technology of Transplantation, Osaka University Graduate School of Medicine, 2-2 Yamadaoka, Suita, Osaka 565-0871, Japan

[2] Department of Nephrology, Osaka University Graduate School of Medicine, 2-2 Yamadaoka, Suita, Osaka 565-0871, Japan

[3] National Institute of Diabetes and Digestive and Kidney Diseases, National Institutes of

Health, Bldg 31, 9A52, 31 Center Drive, Bethesda, MD20892, USA

[4] University of Maryland School of Medicine, Division of Nephrology, 22 S Green St, Baltimore, MD21201, USA

[5] Laboratorio de Investigacion en Nefroloxia, Complexo Hospitalario Universitario de Santiago, Travesía de Choupana, s/n 15706 Santiago de Compostela, Spain

[6] Division of Nephrology, Children's Hospital of Philadelphia, Perelman School of the University of Pennsylvania, 3401 Civic Center Boulevard, CTRB 9207 Philadelphia, PA 19104, USA

[7] Center of Translational Cancer Research and Therapy, State Key Laboratory of Molecular Oncology, Cancer Hospital and Institute, Chinese Academy of Medical Sciences and Peking Union Medical College, Beijing, 100021, China

[8] Graduate School of Pharmaceutical Science, Nagasaki International University, 2825-7 Huis Ten Bosch-Cho, Sasebo, Nagasaki 859-3298, Japan

[9] Departments of Human Pathology, Kanazawa University Graduate School of Medicine, 13-1 Takara-cho, Kanazawa, 920-8640, Japan

[10] Department of Pathology, Shizuoka Cancer Center, 1007 Shimonagakubo, Nagaizumi-cho, Sunto-gun, Shizuoka, 411-8777, Japan

[11] University of Sao Paulo, Department of Medicine, Division of Nephrology, Dr. Arnald Ave, 455-Cerqueira Cesar, Sao Paulo, CEP01246903-903, Brazil

[12] Children's National Health System, 6th Floor Main Hospital, Center 6, 111 Michigan Ave NW, Washington, DC 20010, USA

[13] Johns Hopkins University School of Medicine, Department of Medicine, Division of Nephrology, 1830 East Monument St., Suite 416, Baltimore, MD21287, USA

*: corresponding authors

Jun-ya Kaimori M.D., PhD. Tel: +81-6-6879-3746; Fax: +81-6-6879-3749; E-mail: kaimori@att.med.osaka-u.ac.jp

Gregory G Germino M.D. Tel:301-496-5877; Fax: 301-402-2125; E-mail: germinogg@niddk.nih.gov

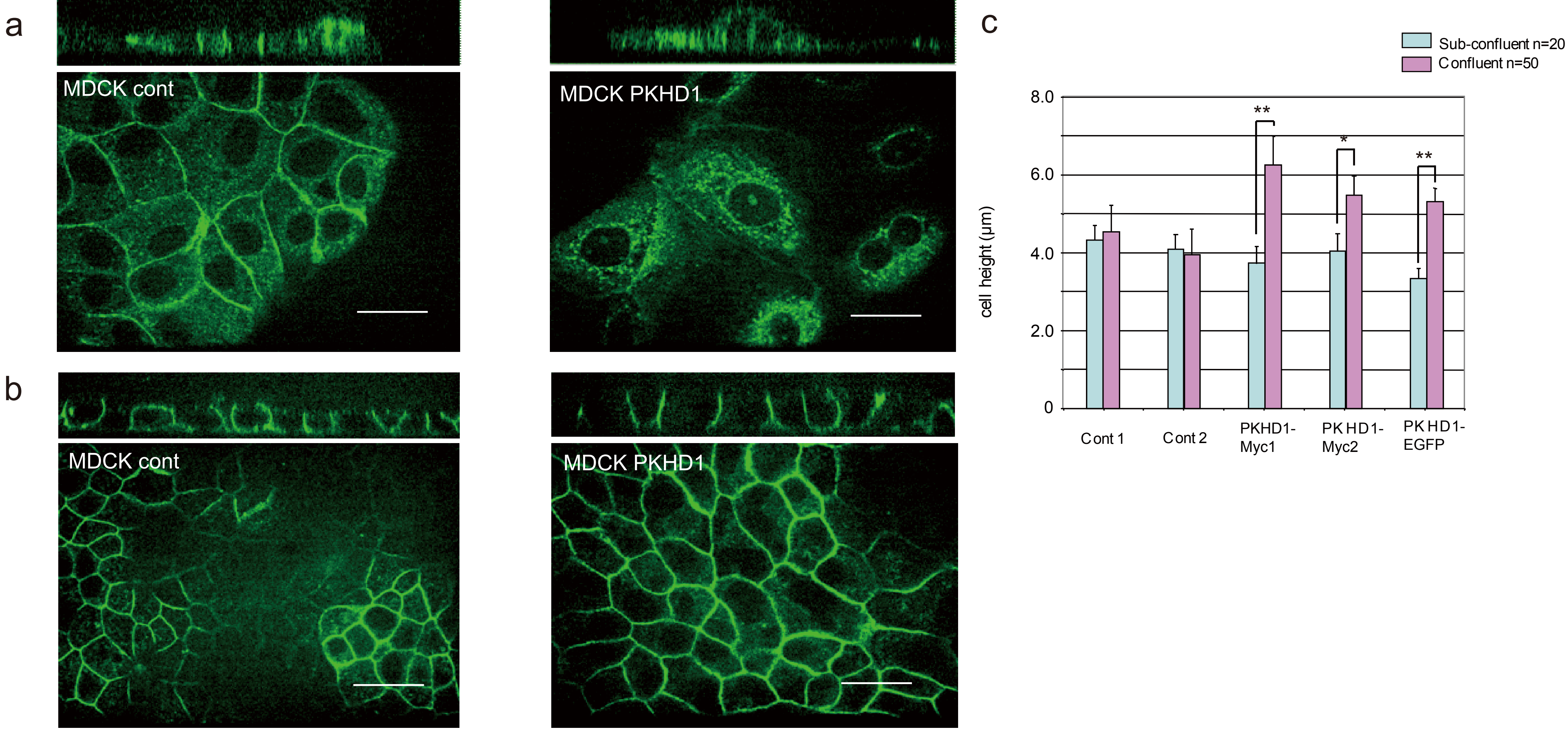

Supplementary Figure S1. Heterologous expression of PKHD1 affects cellular morphology of MDCK cells

a) E-cadherin staining of sub-confluent cultures of Flp-In MDCK cell lines stably transfected with pcDNA5 vector control (left) and human PKHD1 (right). Upper panels are z-stack images of each cell line. Scale bars, 5 µm

b) Same cell lines as in panel "a" cultured to confluence and stained for E-cadherin. Upper panels are z-stack images of each cell line. Scale bars, 20 µm

c) Table comparing heights of low confluent (<40%) and confluent cultures of multiple independently-derived pcDNA5 vector control and PKHD1-expressing Flp-In MDCK cell lines. Heights were measured of z-stack images using LSM image software and values represent mean ± s.d. of 50 (PKHD1 MDCK) and 20 (control) measurements. The height of PKHD1-expressing cell lines increased significantly when cultured to confluence and was higher than that of control cell lines $^{*}P<0.05$, $^{**}P<0.01$.

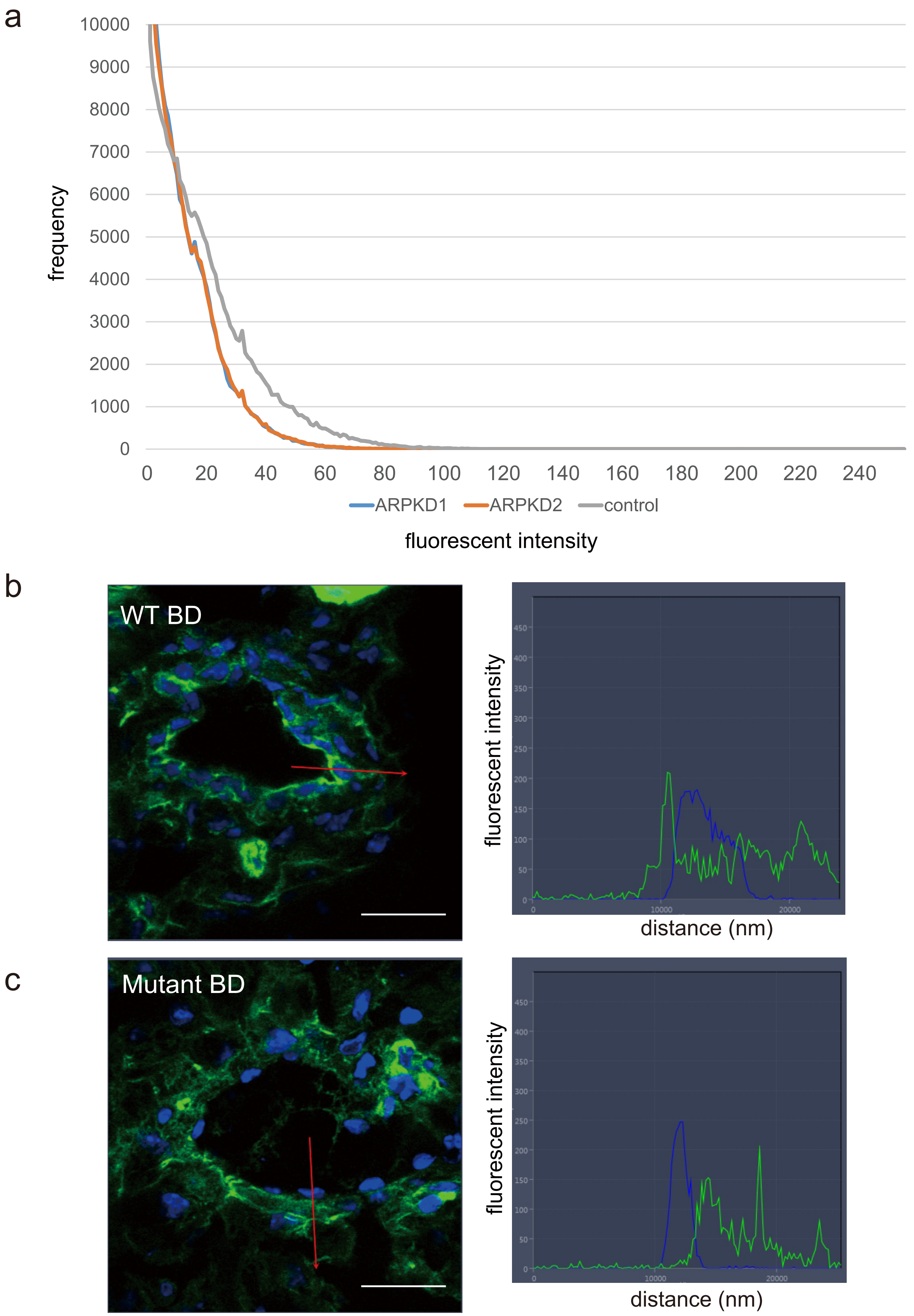

Supplementary Figure S2. Fluorescent intensity analyses of Figure 1f and 1g.
a) Histograms of red fluorescent intensity in Figure 1f.
b), c) Histograms of blue and green fluorescent intensity along the red arrow lines in WT and mutant liver tissues in Figure 1g. Scale bars, 20 µm.

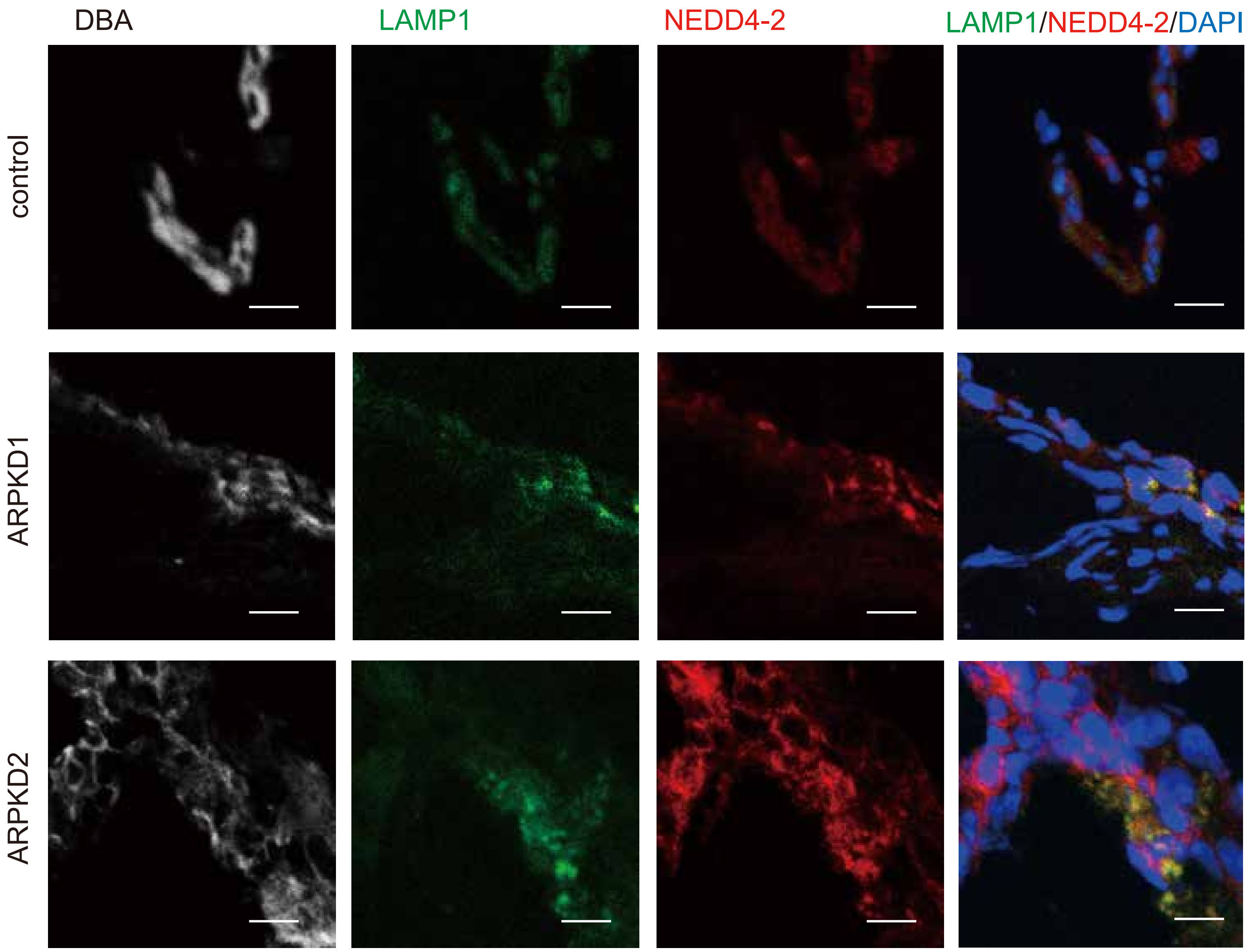

Supplementary Figure S3. NEDD4-2 is mis-localized in human ARPKD kidneys.
Kidney specimens from human control (control) and two different ARPKD donors (ARPKD1, ARPKD2) stained for DBA (grey), LAMP1 (green), DAPI (blue), and NEDD4-2 (red). Scale bars, 10 µm.

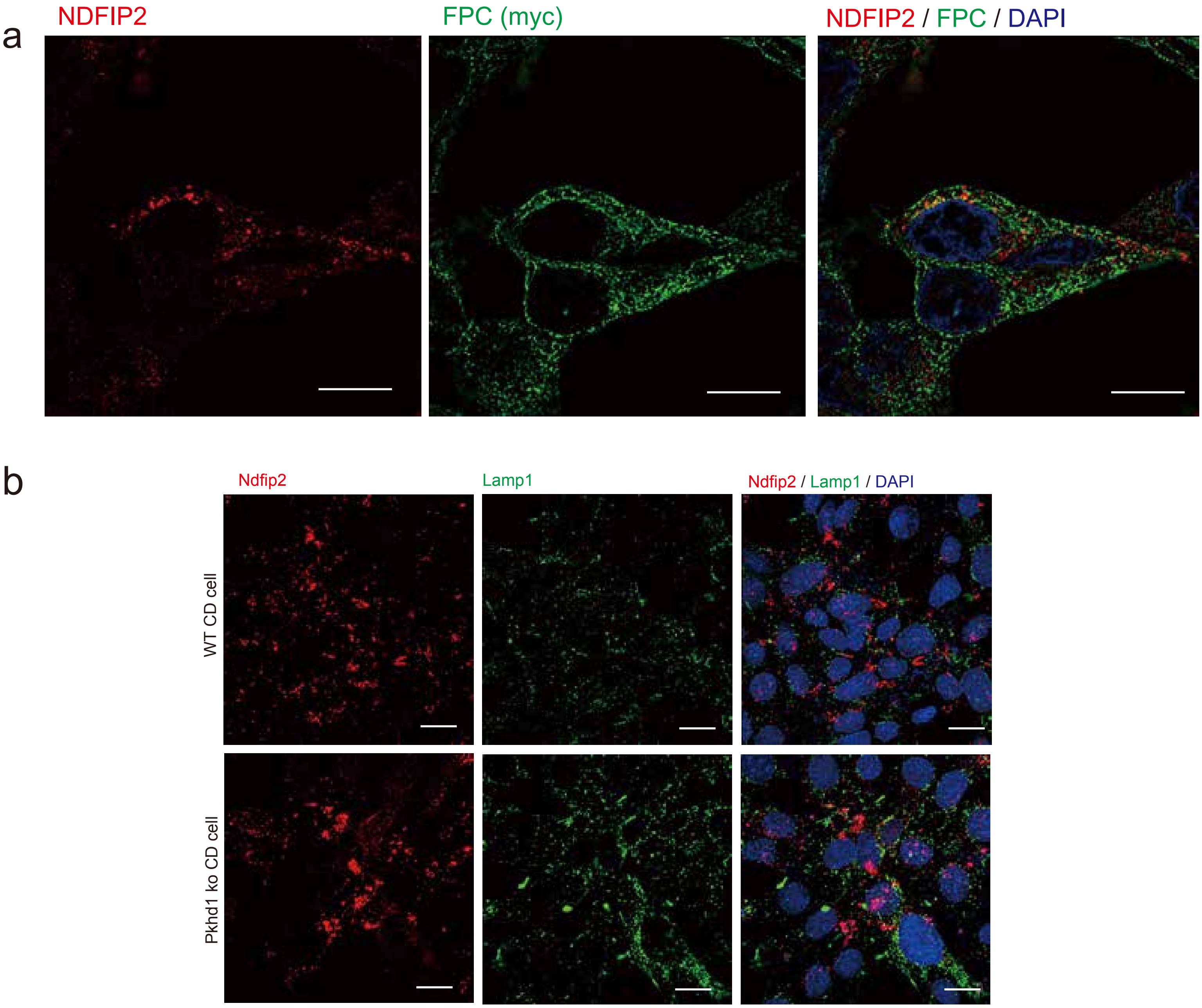

Supplementary Figure S4. NDFIP2 co-localizes with FPC but not Lamp1 in cultured cells.
a) Superresolution images of immunolocalization of endogenous NDFIP2 (red) and FPC (green) in HEK cells expressing Myc-PKHD1. NDFIP2 and FPC co-localize in a subset of small vesicles. Nuclei were stained with DAPI. Scale bar, 10 µm.
b) Immunolocalization of endogenous Ndfip2 (red) and Lamp1 (green) in CD cells derived from wild type and Pkhd1 ko mice. Ndfip2 and Lamp1 localize in distinct subsets of small vesicles in wild type cells. In Pkhd1 mutant cells, Ndfip2 and Lamp1 also localize in distinct subsets of large vesicles. Scale bar, 20 µm.

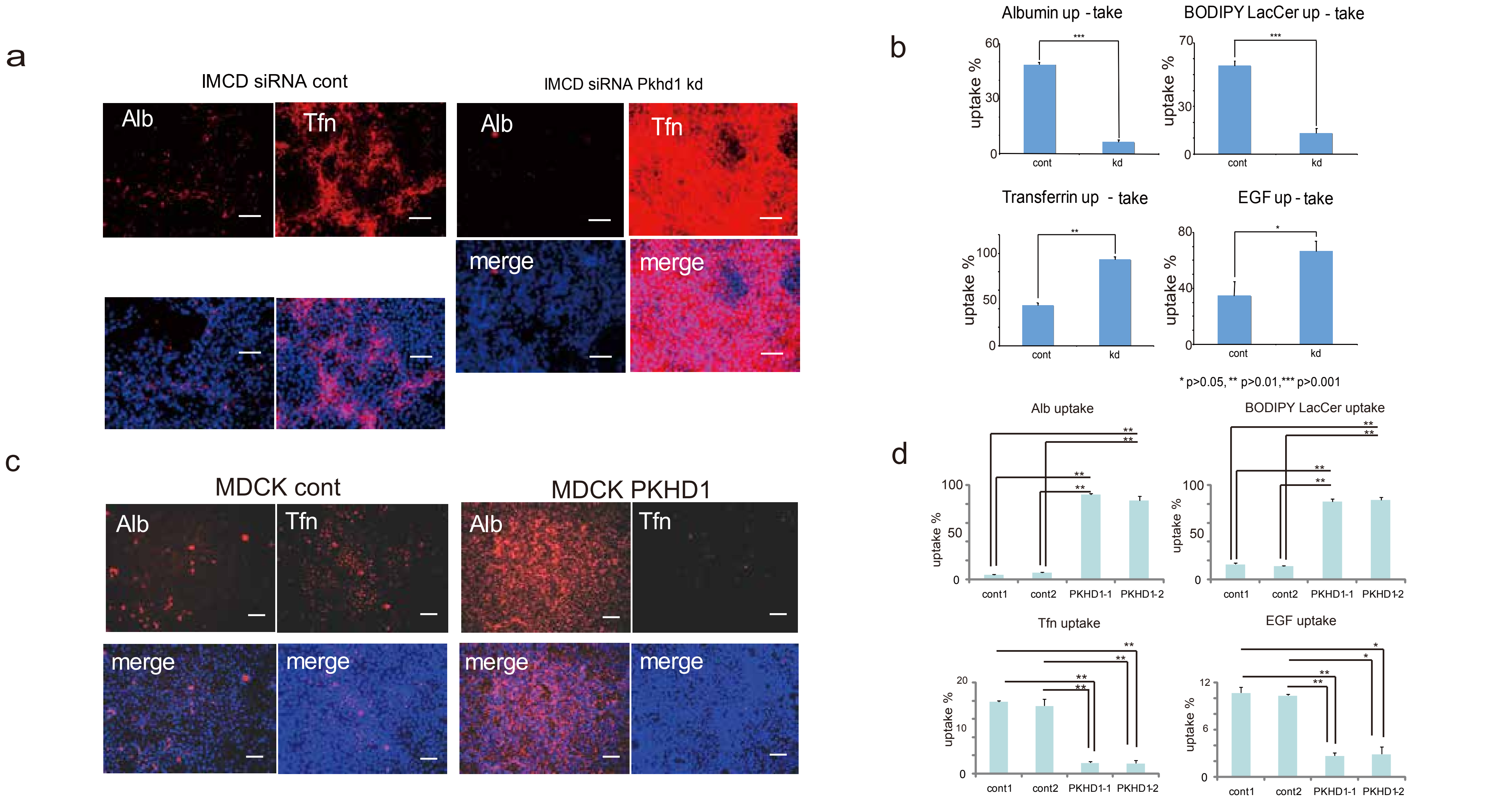

Supplementary Figure S5. Effects of Pkhd1 expression on endocytosis in renal epithelial cell lines.

a) Endocytosis assays in confluent cultures of control or Pkhd1-silenced IMCD cell lines using fluorescently-labeled markers for the caveolar [albumin (Alb, red)] and clathrin [transferrin (Tfn, red)] endocytic pathways. Nuclei were stained with DAPI. Scale bar, 50 μm.

b) Quantitation of results for internalization assays using fluorescent markers for the clathrin (Tfn, EGF) and caveolar (alb, LacCer) endocytic pathways in control and Pkhd1-silenced IMCD cells. Data were obtained using conventional fluorescence and are expressed as percent of uptake. Data represent quantification of at least 100 cells from each of three independent experiments. *P<0.05, ** P<0.01, *** P<0.001.

c) Endocytosis assays in confluent Flp-In MDCK cell lines stably transfected with pcDNA5 vector control (left) and human PKHD1 (right) using fluorescently-labeled albumin (Alb, red) and transferrin (Tfn, red). Nuclei were stained with DAPI (blue). Scale bar, 50 μm.

d) Quantitation of results for internalization assays using fluorescent markers for the clathrin (Tfn, EGF) and caveolar (alb, LacCer) endocytic pathways in two independently-derived Flp-In MDCK control (pcDNA5) lines (cont1, cont2) and two MDCK PKHD1+ cell lines (PKHD1-1, PKHD1-2). Data were obtained using conventional fluorescence and are expressed as percent of uptake. Data represent quantification of at least 100 cells from each of three independent experiments. *P<0.05, ** P<0.01.

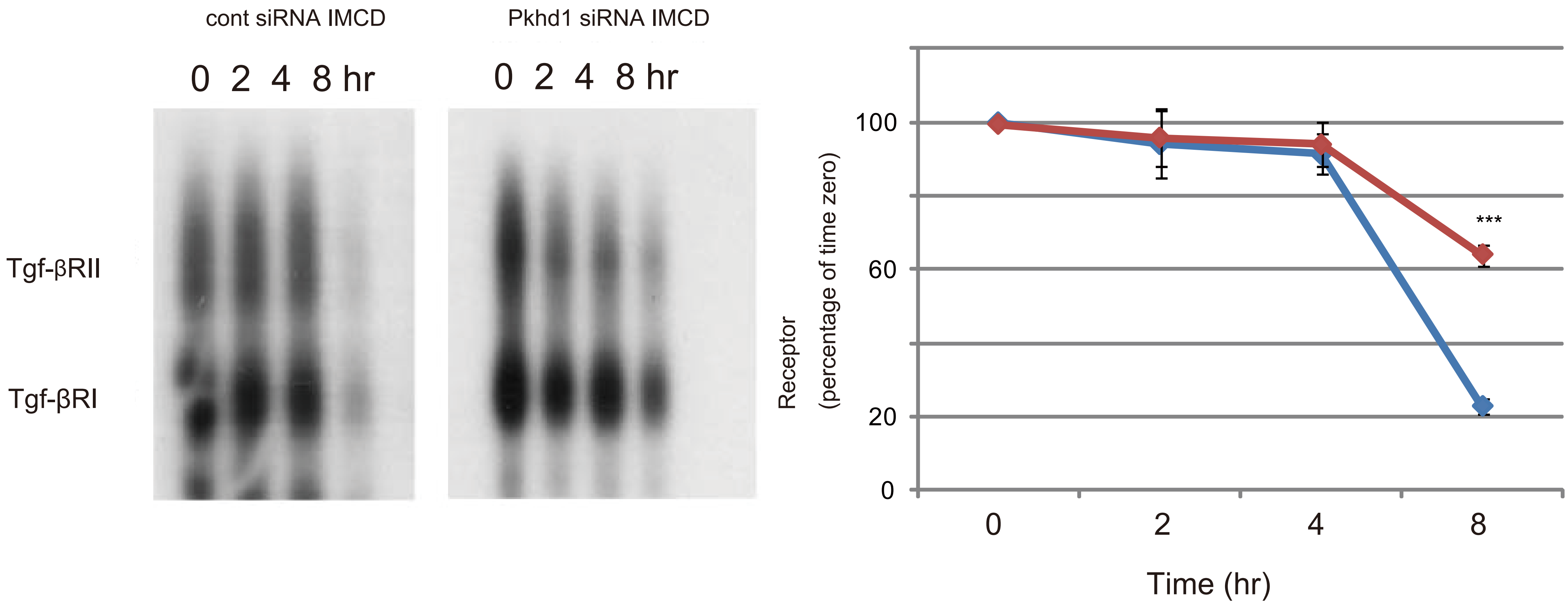

Supplementary Figure S6. Compromised TGF-β receptor degradation in IMCD cell line with siRNA-reduced expression of Pkhd1.
Tgf-β receptor degradation assay in Control and Pkhd1 siRNA IMCD cell lines. On the left, autoradiographs of a representative experiment for control (cont siRNA IMCD) and Pkhd1-silenced (Pkhd1 siRNA IMCD) cell lines treated with [125I]-labeled human TGF-β and then analyzed for levels of labeled Tgf-β receptor subunits at the times indicated. Three separate experiments were carried out, quantified by phosphorimaging and graphed as receptor quantity (% of time 0) vs. time (graph on right). Each point represents the mean ± SD. The results for wild type are in blue and mutant in red. *** P<0.001. The full-length images of these cropped autoradiographs are included in Supplementary Figure S11.

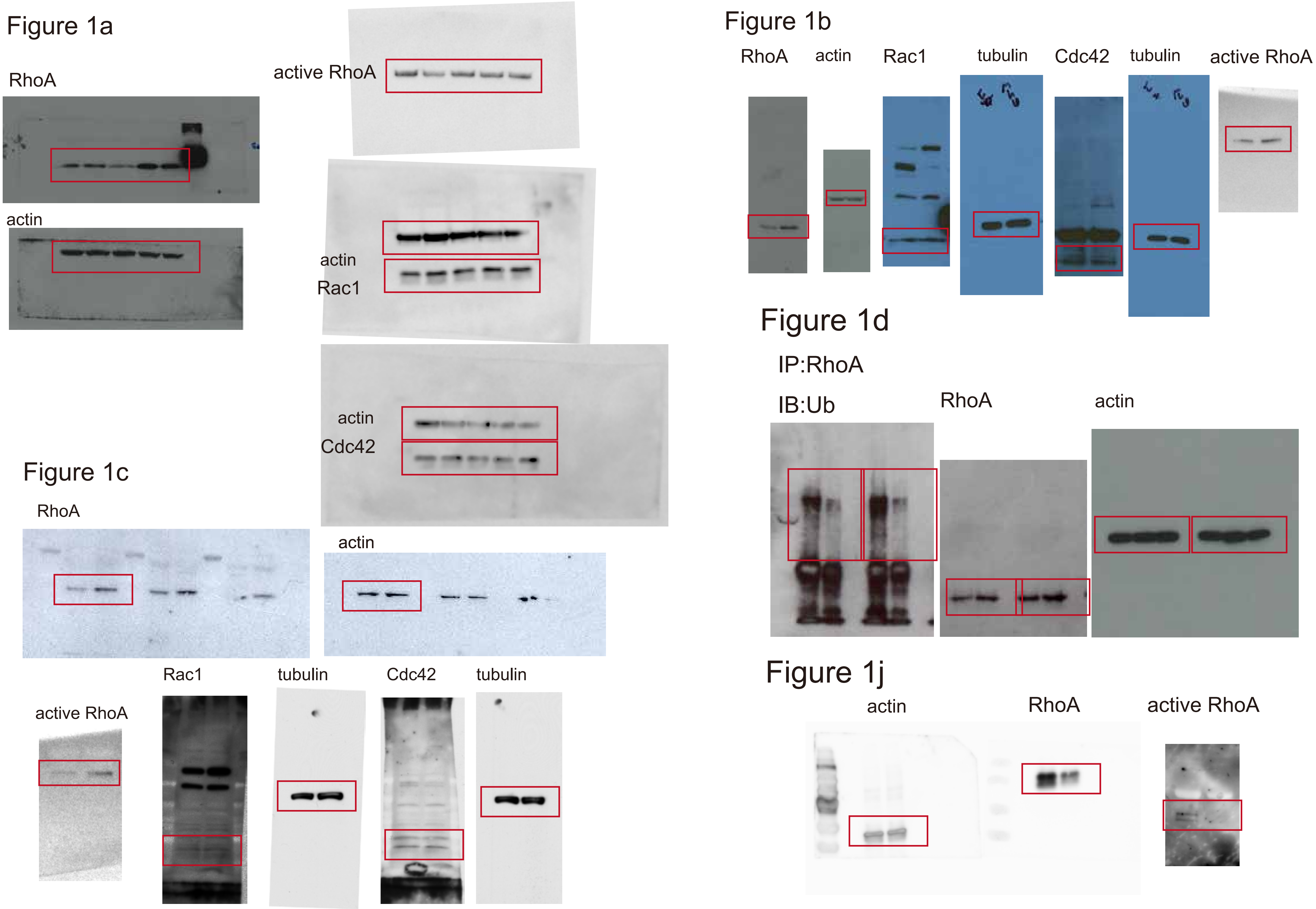

Supplementary Figure S7. Uncropped immunoblots of Figure 1a,b,c,d,j

Figure 2b

Smurf1

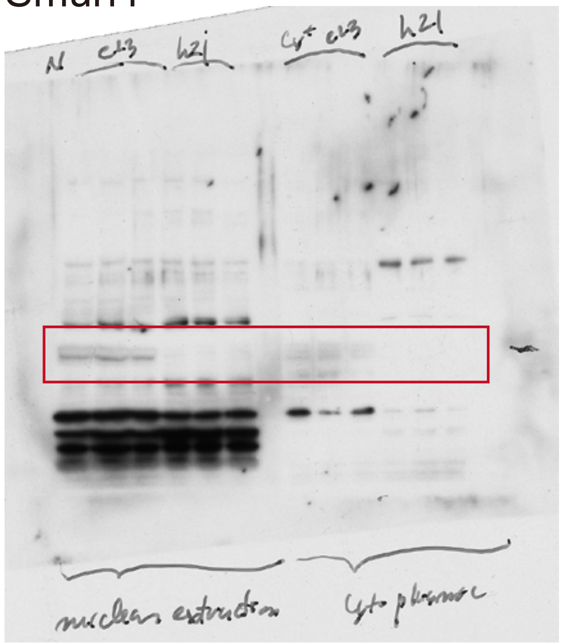

Hdac1

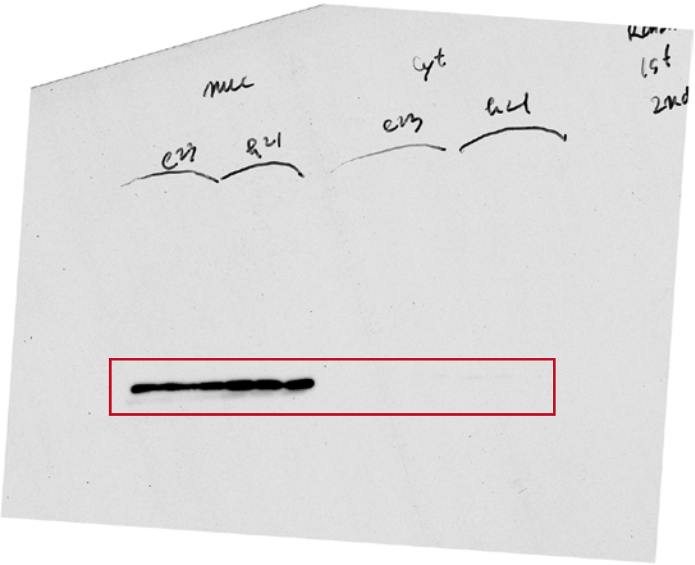

Hsp90/tubulin

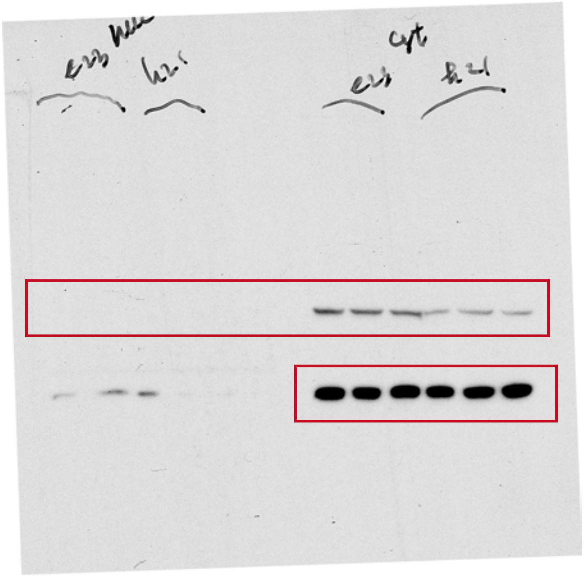

Supplementary Figure S8. Uncropped immunoblots of Figure 2b

Figure 5b

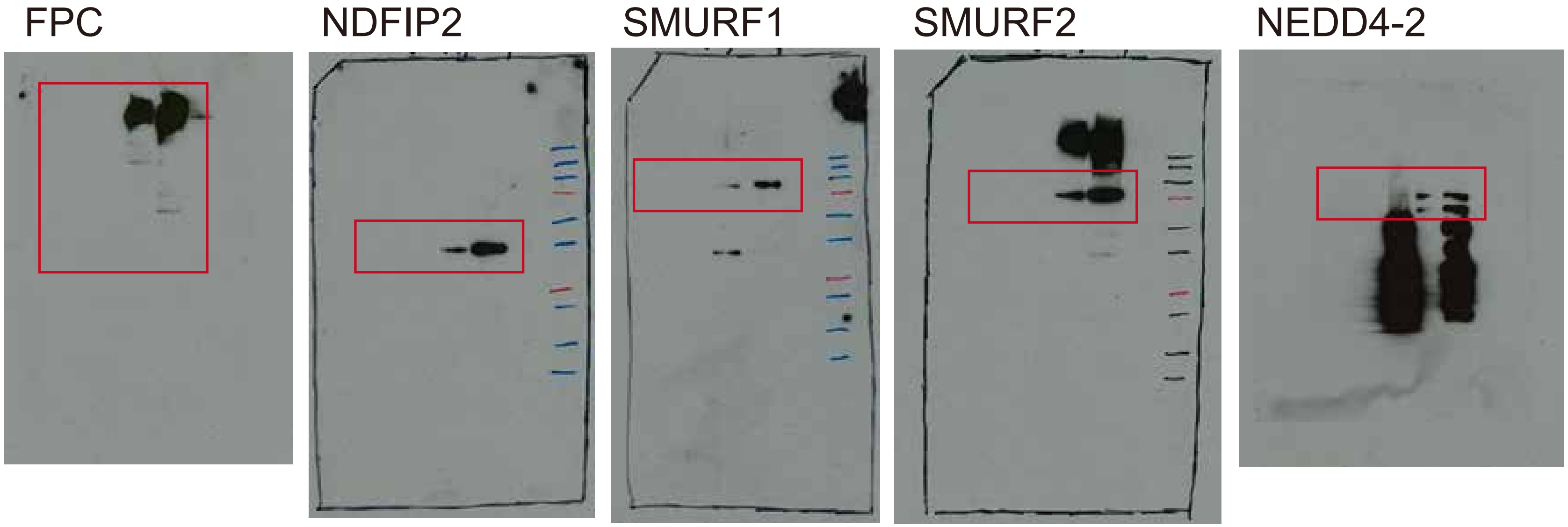

Figure 5c

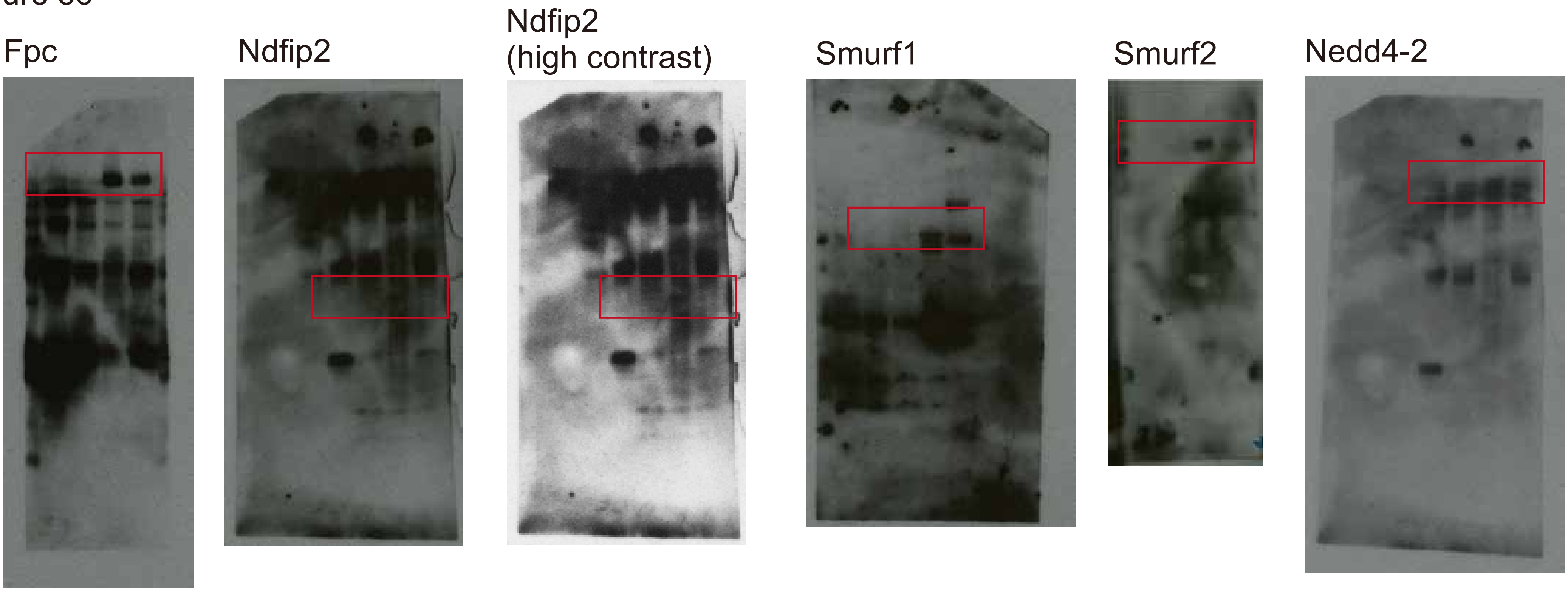

Supplementary Figure S9. Uncropped immunoblots of Figure 5b,c

Figure 6e

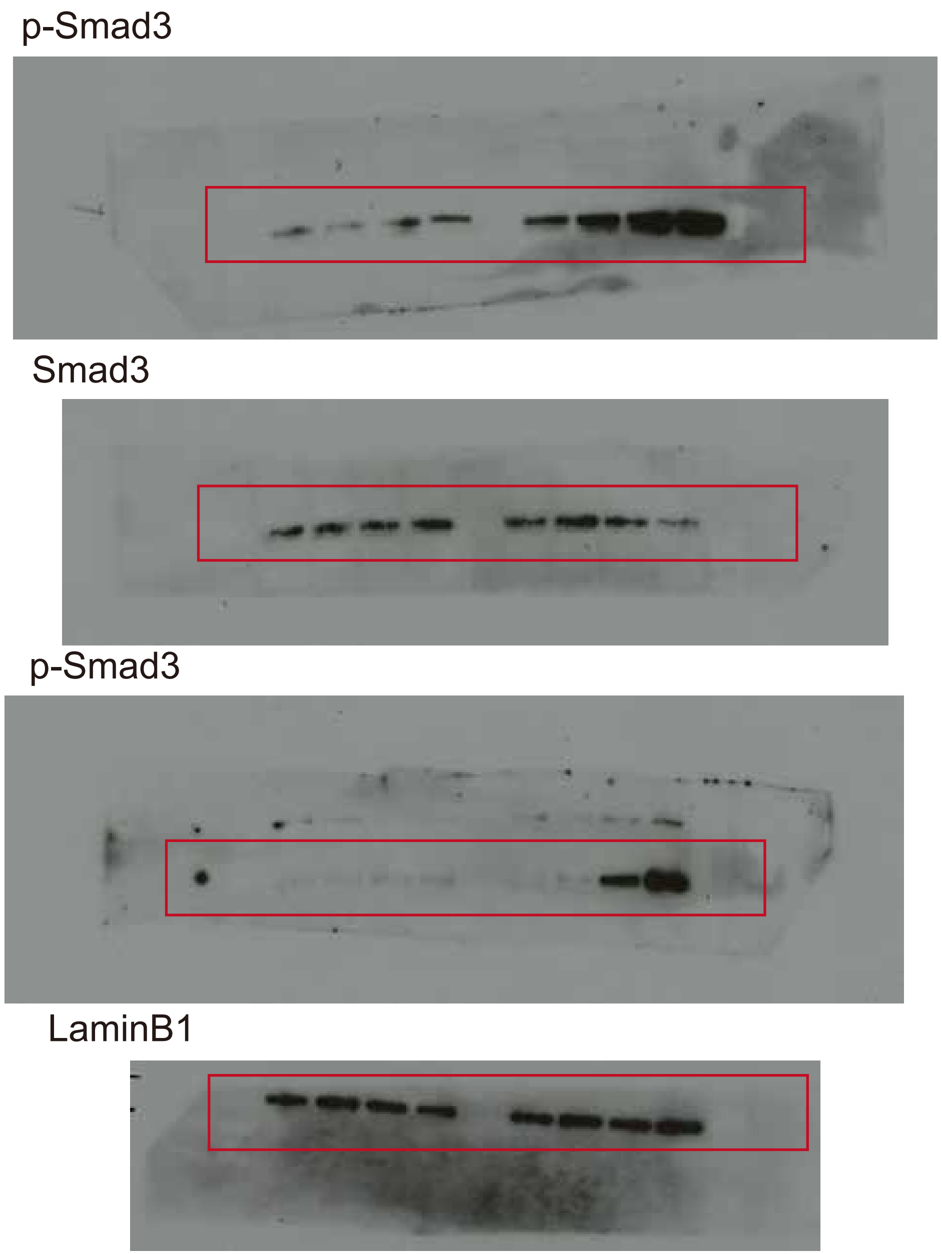

Figure 6f

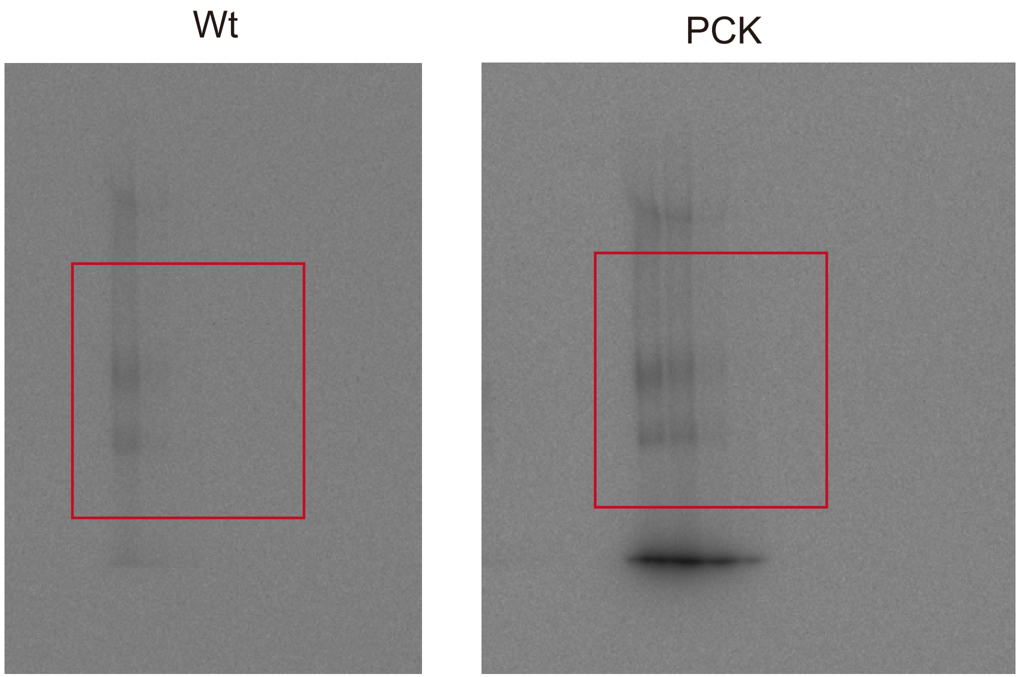

Supplementary Figure S10. Uncropped immunoblots of Figure 6e,f

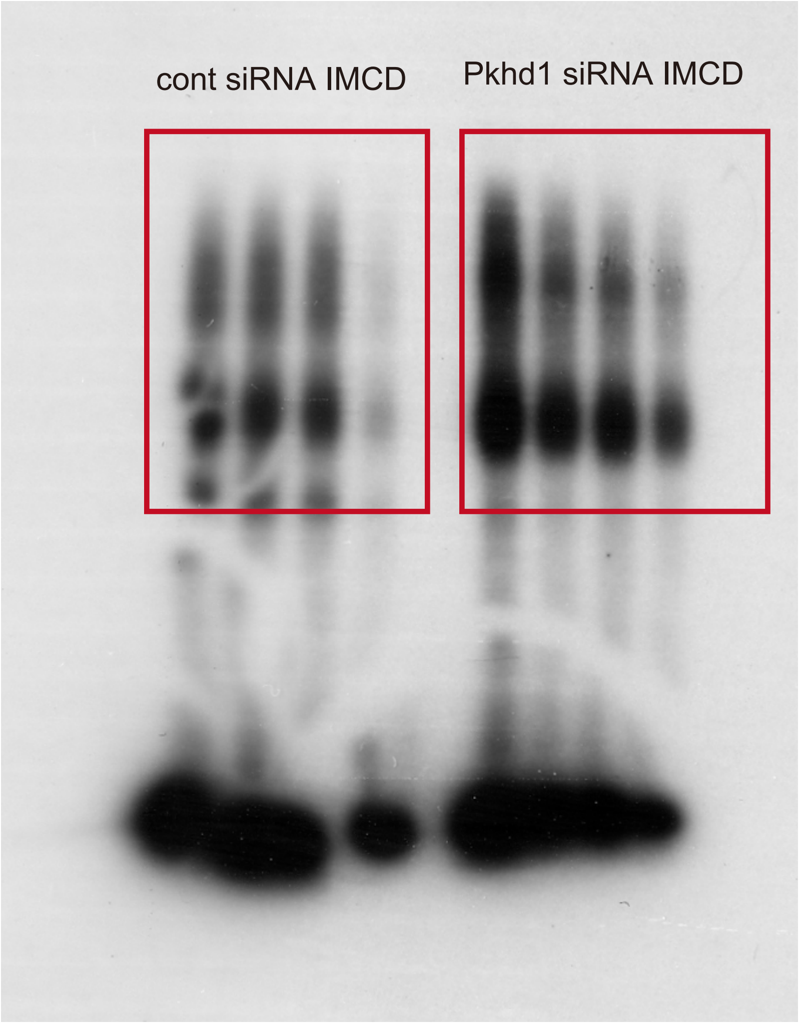

Supplementary Figure S11.Uncropped immunoblots of Supplementary Figure S6
